# Genome-wide identification and characterization of gibberellin metabolic and signal transduction (GA MST) pathway mediating seed and berry development (SBD) in grape (*Vitis vinifera* L.)

**DOI:** 10.1186/s12870-020-02591-1

**Published:** 2020-08-21

**Authors:** Wenran Wang, Yunhe Bai, Padmalatha Koilkonda, Le Guan, Yaxian Zhuge, Xicheng Wang, Zhongjie Liu, Haifeng Jia, Chen Wang, Jinggui Fang

**Affiliations:** 1 Nanjing Agricultural University, College of Horticulture, Nanjing, 210095 PR China; 2 China Agricultural University, College of Horticulture, Beijing, 100193 China; 3 Division of Crop Sciences, ICAR-Central Research Institute for Dryland Agriculture, Santoshnagar, Hyderabad, Telangana 500059 India; 4 Institute of Pomology, Jiangsu Academy of Agricultural Sciences, Nanjing, 210014 China

**Keywords:** Grape berry and seed development, GA MST pathway, Multihormone MST pathway

## Abstract

**Background:**

Grape is highly sensitive to gibberellin (GA), which is crucial during seed and berry development (SBD) either by itself or by interacting with other hormones, such as auxin, Abscisic acid (ABA), and Cytokinin (CK). However, no systematic analysis of GA metabolic and signal transduction (MST) pathway has been undertaken in grapevine.

**Results:**

In this study, total endogenous GA_3_ content significantly decreased during SBD, and a total of 48 known genes in GA metabolic (GAM; 31) and signal transduction (ST; 17) pathways were identified in this process. In the GAM pathway, out of 31 genes, *VvGA20ox1–1*, *VvGA3ox4–1*, and *VvGA2ox1–1* may be the major factors interacting at the green-berry stage (GBS) accompanied with higher accumulation rate. GA biosynthesis was greater than GA inactivation at GBS, confirming the importance of seeds in GA synthesis. The visible correlation between endogenous GA_3_ content and gene expression profiles suggested that the transcriptional regulation of GA biosynthesis pathway genes was a key mechanism of GA accumulation at the stone-hardening stage (SHS). Interestingly, we observed a negative feedback regulation between *VvGA3oxs-VvGAI1–4*, *VvGA2oxs-VvGAI1–4*, and *VvGID1B-VvGAI1–4* in maintaining the balance of GA_3_ content in berries. Moreover, 11 miRNAs may be involved in the modulation of GA MST pathway by mediating their target genes, such as *VvGA3ox*, *VvGID1B*, and *VvGAMYB*. Many genes in auxin, ABA, and CK MST pathways were further identified and found to have a special pattern in the berry, and the crosstalk between GA and these hormones may modulate the complex process during SBD through the interaction gene network of the multihormone pathway. Lastly, based on the expression characterization of multihormone MST pathway genes, a proposed model of the GA-mediated multihormone regulatory network during SBD was proposed.

**Conclusions:**

Our results provided novel insights into GA-mediated regulatory networks during SBD in grape. The complexity of GA-mediated multihormone ST in SBD was also elucidated, thereby providing valuable information for future functional characterizations of specific genes in grape.

## Background

Grape (*Vitis vinifera* L.), which is a perennial fruit crop cultivated globally, is economically important playing a major role for human health served as fresh food, dried food, and beverages and valued for its medicinal importance. The seed and berry development (SBD) is one of the extremely important phases during its life cycle. Grape is highly sensitive to gibberellin (GA), which is one of the essentially significant hormones during SBD. The molecular and genetic analysis in previous studies revealed that GA signal is involved in various stages of berry growth and development [1], such as promoting berry expansion, significantly affecting fruit set, coloring, and development, and ripening of berries. Several studies have demonstrated the exogenous prebloom application of GA_3_-induced seed abortion in the ‘Zuijinxiang’ and ‘Red Globe’ seeded cultivars [2, 3]. Furthermore, GA can increase fruit sink strength [4]; thus, GA can indirectly affect fruit development through affecting energy supplement. During the past two decades, a set of genes involved in GA signal pathway have been identified, and the biological functions of key genes have been extensively characterized in banana [5] and apple [6]. The molecular interaction network of GA signal pathway in regulating SBD at the whole wide-transcriptome level is still unclear in grape.

The control of DELLA stability is a key factor for the GA response and represents a major entry point importance for other phytohormones, such as IAA [7] and ABA [8], affecting plant growth and development. Therefore, we supposed that a network exists between DELLA proteins that mediated GA metabolic and signal transduction (MST) pathways and a cross talk with multihormone MST pathway during SBD. A complete understanding of the GA, ABA, auxin, and CK MST pathways is fundamental for elucidating the mechanisms during grape SBD. With the release of the grape genome sequence (http://genomes.cribi.unipd.it/DATA/V2/V2/) and the increasing affordability of high throughput analysis tools, a better opportunity is present to study the genes related to MST pathways in grape systematically.

The morphological and physiological variation during development of berries obviously implies the expression level variation of many genes, including genes in GA MST pathway. On the basis of the RNA-seq data from four grape berry developmental stages, we identified and characterized the genes involved in GA, auxin, CK, and ABA MST pathways in grape berries and detected their spatio-temporal expression profiles during SBD, together with the accumulation variation of GA_3_, IAA, ZR, and ABA contents along with the berry development, to gain insight into the signal regulatory networks of SBD at the whole wide-transcriptome level. A complex regulatory network of GA-mediating SBD with other hormone cooperative regulations in grape was developed. Our research outcome could enhance understanding and provide valuable information for further elucidation of molecular mechanisms and underlying interactions between GA and ABA, Auxin, CK regulation during SBD.

## Results

### Morphological, physiological, and biochemical variations during SBD

**Fig. 1:**
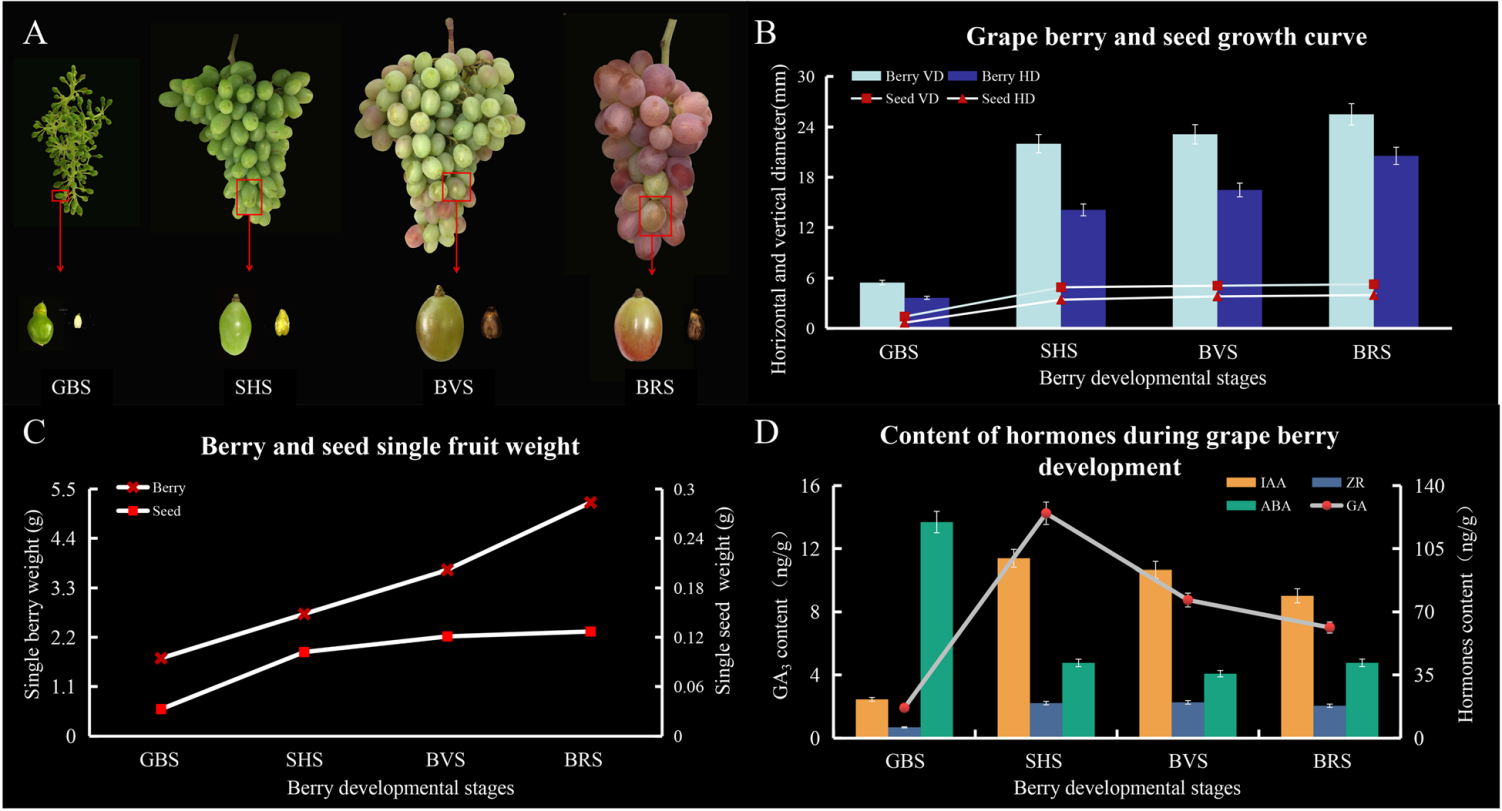
Growth and Variation in berry size during development of berries at four stages (GBS, SHS, BVS and BRS). **a** Color and size variation in clusters, berries and seeds at GBS, SHS, BVS and BRS. **b** Variation in average single seed and berry weights at four developmental stages. **c** Variation in average length and width of single seed and berry during four developmental stages. **d** Endogenous levels of GA_3_, IAA, ZR and ABA during four stages of development of berries

Figure 1a shows that grape berries continued growing from green-berry stage (GBS) until ripening period, whereas grape seeds developed maturity and hardened by SHS. The developmental curve and average weight of berries and seed showed a rapid increase from GBS to SHS, whereas they maintained stability during berry-veraison stage (BVS) and berry-ripening stage (BRS) (Fig. 1b and c). During initial berry growth and GBS, berry size increased along a sigmoidal growth curve due to cell division and expansion. SHS is defined as a lag phase in which cell expansion ceases, seeds harden, embryo rapidly develops, and sugars begin to accumulate. BVS marks the beginning of the BRS in which berries undergo a second period of sigmoidal growth due to additional mesocarp cell expansion, accumulation of anthocyanin, glucose, and fructose, and a decline in organic acid accumulation.

### Contents of endogenous GA_3_, IAA, ZR, and ABA

The levels of endogenous hormones GA_3_, IAA, and ZR during berry development both increased and decreased, whereas the levels of ABA decreased. The highest levels of GA_3_, IAA, and ZR were observed at SHS, whereas ABA was at GBS. The total amount of GA_3_, IAA, and ZR (expressed as ng/g fw) progressively increased from 1.90, 21.38, and 5.81 ng/g fw at GBS to 14.24, 99.62, and19.28 ng/g fw at SHS, and decreased from to 7.00, 78.93, and 17.84 ng/g fw at BRS (Fig. 1d). ABA was detected at 119.73 ng/g fw at GBS to 41.67 ng/g fw in ripe berry.

### Identification of genes in GA MST pathway

In grape, a total of 48 non-redundant GA MST-related genes were identified in this study. Each of them was named based on the enzymatic reaction and referred to *Arabidopsis thaliana* GA MST pathway. Among these 48 GA MST-related genes, 31 and 17 genes were involved in GAM and ST, respectively. These genes belonged to nine different gene families, and all of them were identified as single gene copy. Two genes showed no change in their expression levels during SBD, including *VvGA20ox1–2* and *VvGA20ox2–1*, and 2 transcripts were observed as upregulated or downregulated slightly. About 44 transcripts were considered as significantly differentially expressed genes (Tables 1 and 2).

**Table 1:** GA Metabolic pathway genes in grape berry

Name	Accession no.	Family	Chrom	Gene start	Gene end	Gene length	Gene strand
*VvGA20ox1-B1*	VIT_215s0046g02550	2OG-Fe (II) oxygenase	chr15	19,336,280	19,337,354	987	+
*VvGA20ox1-B2*	VIT_216s0050g00640	2OG-Fe (II) oxygenase	chr16	17,592,866	17,594,861	984	–
*VvGA20ox1–1*	VIT_203s0063g01150	2OG-Fe (II) oxygenase	chr3	4,642,814	4,644,285	1029	–
*VvGA20ox1–2*	VIT_202s0234g00010	2OG-Fe (II) oxygenase	chr2	11,691	13,858	1074	–
*VvGA20ox1–3*	VIT_209s0002g05290	2OG-Fe (II) oxygenase	chr9	5,011,954	5,013,413	936	–
*VvGA20ox2–1*	VIT_218s0001g01390	2OG-Fe (II) oxygenase	chr18	1,982,611	1,985,550	1137	–
*VvGA20ox2–2*	VIT_215s0048g01320	2OG-Fe (II) oxygenase	chr15	15,453,736	15,455,160	1155	+
*VvGA20ox2–3*	VIT_204s0044g01520	2OG-Fe (II) oxygenase	chr4	23,110,636	23,112,150	1128	–
*VvGA20ox2–4*	VIT_204s0044g01650	2OG-Fe (II) oxygenase	chr4	23,382,366	23,384,136	1134	–
*VvGA20ox3–1*	VIT_209s0002g05280	2OG-Fe (II) oxygenase	chr9	5,008,806	5,010,211	945	–
*VvGA20ox3–2*	VIT_209s0002g05320	2OG-Fe (II) oxygenase	chr9	5,054,916	5,056,221	942	–
*VvGA20ox*	VIT_216s0022g02310	2OG-Fe (II) oxygenase	chr16	14,861,534	14,863,137	1149	–
*VvGA3ox1–1*	VIT_209s0002g05340	2OG-Fe (II) oxygenase	chr9	5,075,023	5,077,440	951	–
*VvGA3ox1–2*	VIT_204s0008g04940	2OG-Fe (II) oxygenase	chr4	4,431,414	4,432,859	1068	–
*VvGA3ox1–3*	VIT_209s0002g05300	2OG-Fe (II) oxygenase	chr9	5,037,560	5,040,878	1080	–
*VvGA3ox1–4*	VIT_209s0002g05270	2OG-Fe (II) oxygenase	chr9	4,993,458	4,995,066	1098	+
*VvGA3ox3*	VIT_209s0002g05350	2OG-Fe (II) oxygenase	chr9	5,095,320	5,096,737	954	–
*VvGA3ox4–1*	VIT_204s0008g04920	2OG-Fe (II) oxygenase	chr4	4,415,690	4,418,033	951	+
*VvGA3ox4–2*	VIT_204s0044g02010	2OG-Fe (II) oxygenase	chr4	23,794,890	23,796,653	897	–
*VvGA3ox4–3*	VIT_202s0025g03440	2OG-Fe (II) oxygenase	chr2	2,939,781	2,941,071	966	+
*VvGA2ox1–1*	VIT_219s0140g00120	2OG-Fe (II) oxygenase	chr19	15,497,747	15,499,547	972	+
*VvGA2ox1–2*	VIT_213s0067g01150	2OG-Fe (II) oxygenase	chr13	635,013	637,333	1149	+
*VvGA2ox1–3*	VIT_219s0140g00140	2OG-Fe (II) oxygenase	chr19	15,603,314	15,604,866	999	+
*VvGA2ox2–1*	VIT_205s0077g00520	2OG-Fe (II) oxygenase	chr5	343,963	346,779	1020	–
*VvGA2ox2–2*	VIT_207s0005g01920	2OG-Fe (II) oxygenase	chr7	4,377,229	4,379,446	1002	+
*VvGA2ox2–3*	VIT_209s0002g05310	2OG-Fe (II) oxygenase	chr9	5,051,513	5,052,701	813	–
*VvGA2ox8–1*	VIT_201s0010g01650	2OG-Fe (II) oxygenase	chr1	17,965,019	17,969,123	1092	–
*VvGA2ox8–2*	VIT_219s0177g00030	2OG-Fe (II) oxygenase	chr19	5,864,515	5,868,916	1104	–
*VvGA2ox8–3*	VIT_210s0116g00410	2OG-Fe (II) oxygenase	chr10	190,070	195,160	1035	–
*VvGA2ox8–4*	VIT_206s0004g06790	2OG-Fe (II) oxygenase	chr6	7,476,208	7,478,569	1014	+
*VvGA2ox*	VIT_210s0003g03490	2OG-Fe (II) oxygenase	chr10	5,846,605	5,848,332	1002	+

**Table 2:** GA Signal transduction pathway genes in grape berry

Name	Accession no.	Family	Chrom	Gene start	Gene end	Gene length	Gene strand
*VvGID2–1*	VIT_207s0129g01000	F-box	chr7	16,134,715	16,135,491	555	+
*VvGID2–2*	VIT_218s0001g09700	F-box	chr18	8,077,244	8,078,167	555	+
*VvGID1B*	VIT_207s0104g00930	GID1	chr7	2,061,408	2,063,888	1035	–
*VvGAI1–1*	VIT_214s0068g01610	GRAS	chr14	25,316,395	25,318,488	2007	–
*VvGAI1–2*	VIT_217s0000g10300	GRAS	chr17	12,556,730	12,558,304	1560	–
*VvGAI1–3*	VIT_207s0005g01500	GRAS	chr7	4,026,350	4,028,863	1815	–
*VvGAI1–4*	VIT_214s0006g00640	GRAS	chr14	14,806,872	14,809,122	1710	+
*VvGAI1–5*	VIT_201s0011g05260	GRAS	chr1	4,895,037	4,897,415	1773	–
*VvRGL1*	VIT_219s0085g00540	GRAS	chr19	22,922,082	22,923,674	1593	+
*VvSLR1*	VIT_211s0016g04630	GRAS	chr11	3,959,481	3,961,177	1599	–

### In silico characterization of genes in GA MST pathway during berry development

#### Chromosomal distribution and collinearity of genes

**Fig. 2:**
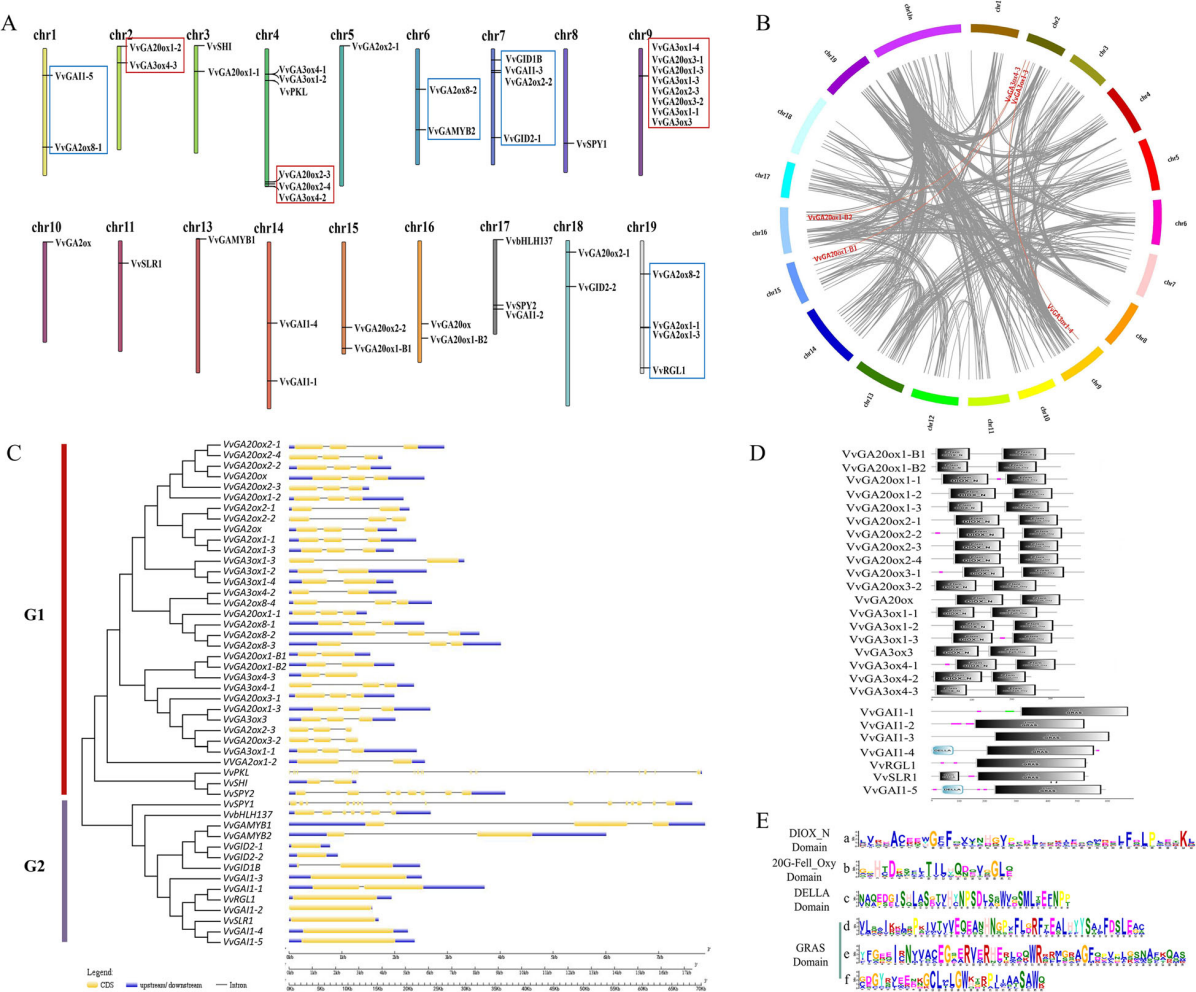
Chromosomal locations, Sequence feature analysis, gene duplication and phylogenetic analysis of GA MST pathway genes. **a** Chromosomal location of GA MST pathway genes. **b** Synteny analysis of GA MST pathway genes. Gray lines indicate all synteny blocks in the grape genome whereas the red lines suggest duplicated gene pairs. **c** NJ phylogenetic tree of GA MST pathway proteins and exon-intron structure of GA MST pathway genes. Three different legends were used 2 genes with long genomic sequence (*VvPKL* and *VvSPY1*) used 70 kb and 17 kb legends, and the others genes used the short legends. Yellow indicates exons; black indicates introns; blue indicated UTRs. **d** Domains of *VvGA20oxs* and *VvGA3oxs* family and DELLA proteins. **e** The DIOX_N domain and 20G-Fell_Oxy domain sequences are highly conserved across all GA20ox and GA3ox proteins, and GRAS domain are highly conserved in DELLA proteins, while DELLA domain is only in SLR, GAI1–4 and GAI1–5 protein. The height of the letter (amino acid) at each position represents the degree of conservation. (Color figure online)

A total of 48 genes in GA MST pathway could be assigned to 18 out of 19 grape chromosomes (except for Chr 2), and uneven distribution of genes across the chromosomes was observed. Chr9 harbored most of the genes (8), followed by Chr4 (6), Chr7 (4), Chr19 (4), Chr17 (3), and the remaining Chrs with 1–2. The results revealed that the genes, such as*VvGA20oxs* and *VvGA3oxs*, in the GA biosynthesis pathway tended to localize on the same Chrs, that is, on Chr 4 and 9, whereas *VvGA2oxs* in GA inactive pathway were localized on the same Chrs, that is, Chr1, 7, and 19, also the genes in GA signal pathway such as *VvGID1B*(Chr7), *VvGAIs* (Chr1, 7, 14, and 17), and *VvRGL1* (Chr9). These results indicated that similar functional genes may possess certain conservation in their locus in grape genome (Fig. 2a).

Furthermore, 12 genes (i.e., *VvGA3ox4–1*/*VvGA3ox1–2*, *VvGA3ox1–4*/*VvGA20ox3–1*/*VvGA20ox1–3*/*VvGA3ox1–3*/*VvGA2ox2–3*/*VvGA20ox3–2*/*VvGA3ox1–1*/*VvGA3ox3*, and *VvGA2ox1–1*/*VvGA2ox1–3*) were clustered into three tandem duplication event regions on grape Chr4, 9, and 19 (Fig. 2b). Besides the tandem duplication events, three segregation duplication events (i.e., *VvGA20ox1-B1*/*VvGA3ox4–3*, *VvGA20ox1-B2*/*VvGA3ox4–3*, and *VvGA3ox1–4*/*VvGA3ox1–3*) were also identified (Fig. 2b), indicating that some grape genes in GA MST pathway were possibly generated by gene duplication. Moreover, the segregation duplication events can also provide a reference for the GA MST gene evolutionary relationship and functional prediction.

#### Phylogenetic tree and exon–intron organization of genes in GA MST pathway

The phylogenetic tree classified the proteins coded by genes in the GA MST pathway into two groups, G1 and G2 (Fig. 2c). All genes in GA metabolism pathway were clustered into G1, whereas except for *VvPKL*, *VvSHI*, and *VvSPY2*, the remaining genes were clustered in GA ST pathway into G2.

Unlike their locations in Chrs, *VvGA20ox*s was closer to *VvGA2oxs* than *VvGA3oxs*. Further analysis of the exon–intron structure on the genes in GA MST pathway showed that similar to their phylogenetic tree, the exon numbers of the majority of genes in G1 were 3 or 2, whereas those in G2 were 1 (Fig. 2d). However, high variation was observed in the number of exons and introns in all genes in GA MST pathway. Eight genes (i.e., *VvGID2–1*, *VvGID2–2*, *VvGAI1–3*, *VvRGL1*, *VvGAI1–2*, *VvSLR1*, *VvGAI1–4*, and *VvGAI1–5*) had no intron in the grape genome; whereas *VvPKL* was the longest gene with 70 kb genomic sequence in GA MST pathway and contained the most exons (29) in this study, followed by *VvSPY1* with 16 exons, *VvSPY2* and *VvbHLH137* with 8/7, respectively, whereas the remaining 42 genes all had 1–3 exons (Fig. 2d).

#### Features of conserved domains and motifs

The DIOX_N domain and 20G-Fell_Oxy domain are common domains of 20 *VvGA20oxs* and *VvGA3oxs*. On the basis of the same domains, we identified the highly conserved motif of DIOX_N domain (Fig. 2. F-a) and 20G-Fell_Oxy domain (Fig. 2. F-b). Additionally, *VvGA20oxs* and *VvGA3oxs* were localized on the same Chrs. Similar to *VvGA20oxs* and *VvGA3oxs*, three highly conserved motifs of GRAS domain were identified in seven DELLA proteins. DELLA domain was found in *VvGAI1–4*, *VvSLR1*, and *VvGAI1–5.* Furthermore, they clustered into the same branch of phylogenetic tree, suggesting they may have similar function.

### Expression analysis of genes in GA MST pathway

#### Biosynthesis pathway

**Fig. 3:**
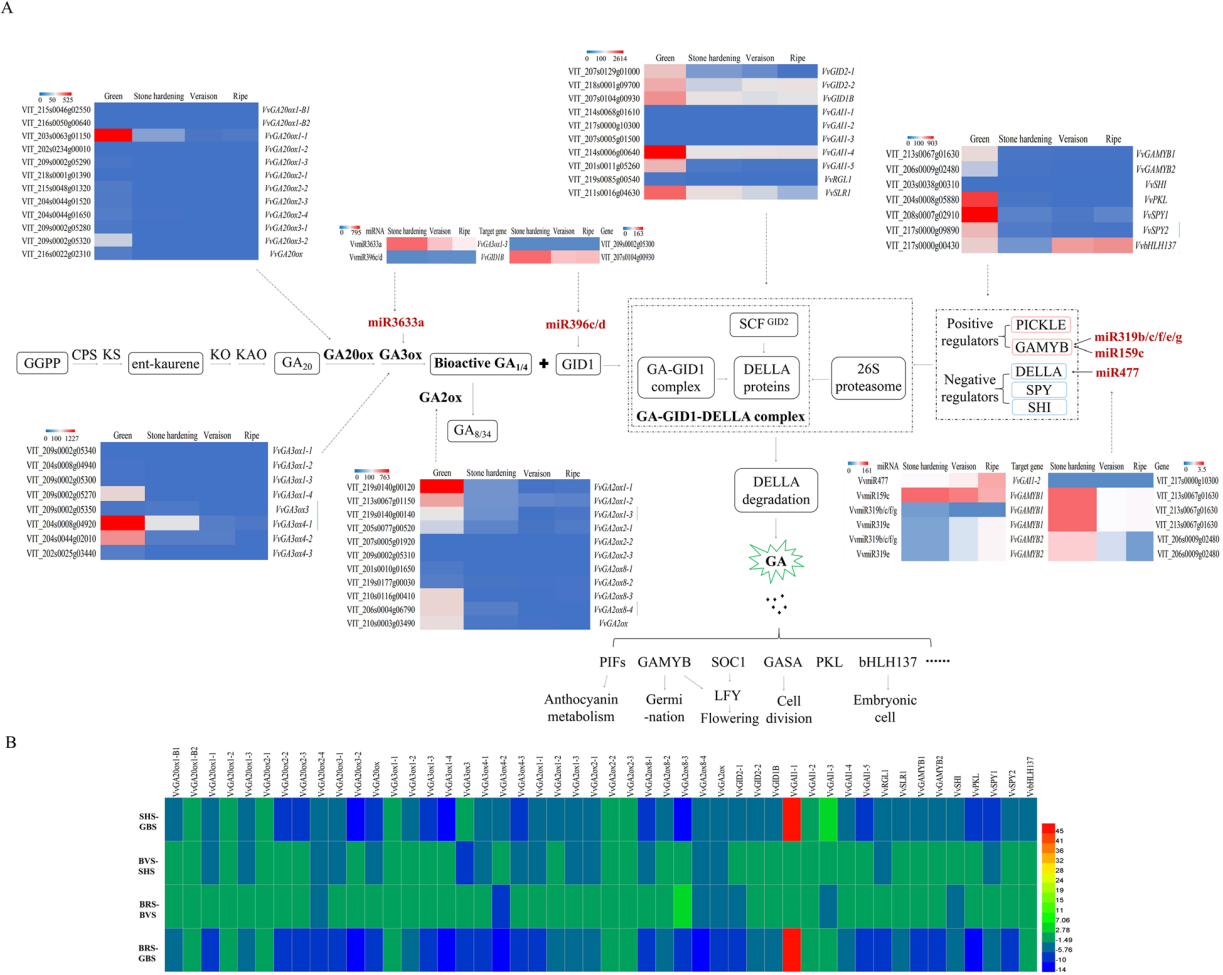
Expression of GA MST pathway genes during berry development. **a** Gene expression was measured by RPKM (reads per kilo base of exon model per million reads). **b** Heat map showing changes in transcript levels of GA MST pathway genes expressed in berries, which was measured by log2 Ratio

In this study, 20 genes, including 12 GA20ox and 8 GA3ox, were involved in GA biosynthesis pathway (Fig. 3a and b; Additional file 1: Table S1), are particularly important for control of bioactive GA levels. Among 12 *VvGA20oxs*, *VvGA20ox1–1* showed the highest expression levels at GBS with significant expression levels in all four stages. *VvGA20ox1-B1*, *VvGA20ox1-B2*, *VvGA20ox1–2*, and *VvGA20ox2–1*showed no expression during SBD. Some members of *VvGA20oxs* family showed significantly lower expression in *VvGA20ox1–3*, *VvGA20ox2–2*, *VvGA20ox2–4*, *VvGA2ox3–1*, and *VvGA20ox*. Similarly, among the 8 members of another significant enzyme GA3ox family, the expression of *VvGA3ox4–1* was higher than that of *VvGA3ox4–2* and *VvGA3ox1–4*. They showed the highest expression at GBS of berries, and its expression significantly decreased from GBS to BRS down to the lowest level, whereas other members only had slight or no expression during SBD*.*

#### Inactivation pathway

*VvGA2oxs* is an enzyme used in GA inactivation pathway. Here, 11 *VvGA2ox* genes were identified in berries. *VvGA2ox1–1*, of which *VvGA2ox1–1*, *VvGA2ox2–1*, *VvGA2ox8–3*, *VvGA2ox8–4*, and *VvGA2ox*, exhibited the highest expression and showed significant variation during the four stages of berry development (Fig. 3a and b; Additional file 1: Table S1). They had the highest expression at GBS. Nevertheless, *VvGA2ox2–2*, *VvGA2ox2–3*, *VvGA2ox8–1*, and *VvGA2ox8–2* were almost not expressed or changed in the four stages of berry development.

#### Signal transduction pathway

One *VvGID1B* gene (GA receptor) was identified and had the highest and lowest expression levels at GBS and BVS, respectively (Fig. 3a). We identified the two members of GID2 family in grape berries that exhibited the expression peak at GBS. *VvGID2–1* gradually decreased from the SHS to BRS, whereas *VvGID2–2* exhibited the lowest expression at SHS. We identified seven genes of DELLA family including *VvGAI1–1*, *VvGAI1–2*, *VvGAI1–3*, *VvGAI1–4*, *VvGAI1–5*, *VvRGL1*, and *VvSLR1*. *VvGAI1–4* had the highest expression, followed by *VvSLR1* and *VvGAI1–5*, and the remaining genes had lower expressions through grape berry developmental process; *VvGAI1–4*, *VvSLR1*, and *VvGAI1–5* predominantly expressed especially at GBS (Fig. 3a and b; Additional file 1: Table S1).

#### Regulatory factors

We revealed that two positive action components *VvGAMYB* and *VvPKL* were primarily expressed at GBS in berries, whereas another four negative action components *VvSPY1*, *VvSPY2*, and *VvbHLH137* were also detected in berries at four stages, and only *VvSHI* possessed low expression levels throughout the four stages in berries (Fig. 3a and b; Additional file 1: Table S1). A total of 10 VvmiRNAs, such as *VvGA3oxs* for VvmiR3633a, *VvGIB1B* for VvmiR396c/d, *VvGAI1–2* for VvmiR477, *VvGAMYB1* for VvmiR159c and VvmiR319b/c/e/f/g, and *VvGAMYB2* for VvmiR319b/c/e/f/g, may be involved in the modulation of GA signal pathway by mediating their target genes. These VvmiRNAs and their target genes exhibited the opposite expression trends in berries, indicating that these VvmiRNAs possessed negative regulatory roles on these potential target genes during SBD (Fig. 3a and b; Additional file 1: Table S1). Moreover, among these VvmiRNAs, VvmiR3633a had the highest expression in SHS berries, whereas the remaining ones were slightly expressed in berries. The target gene *VvGID1B* for VvmiR396c/d exhibited the highest expression also at SHS, implying that VvmiR3633a may repress GA biosynthesis at SHS by modulating the expression level of *VvGA3ox1–3*. Given that the VvmiR396c/d had low expression level, resulting into the high expression of *VvGID1B* may strengthen the GA signal receptivity.

### Expression characterization of genes in multihormone MST pathways

**Fig. 4:**
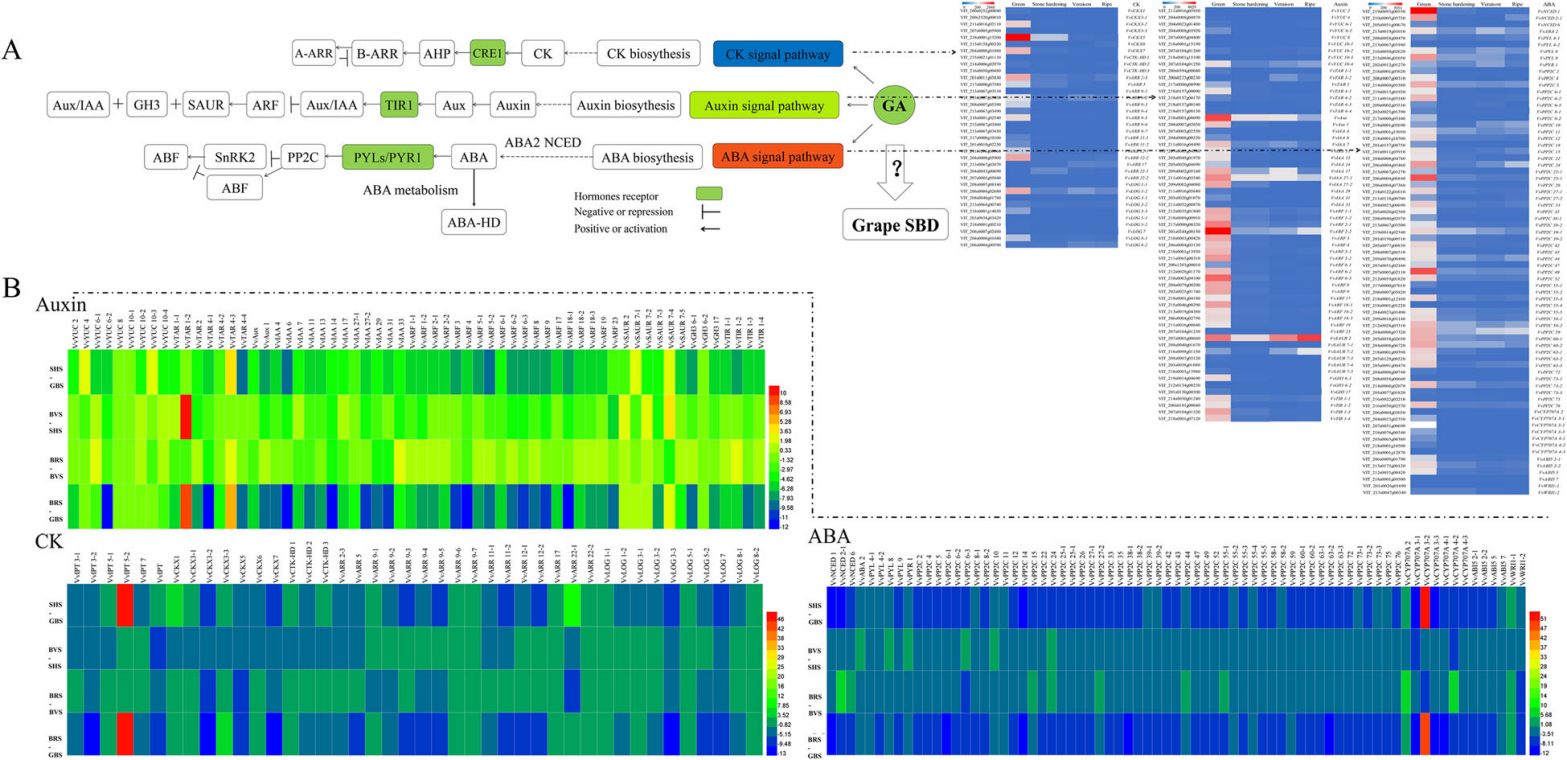
The crosstalk of GA with other hormones, like auxin, ABA, and CK. **a** Gene expression was measured by RPKM (reads per kilo base of exon model per million reads). **b** Heat map showing changes in transcript levels of hormones MST pathway genes expressed in berries, which was measured by log2 Ratio

From our dataset, we also identified the 62, 36, and 73 genes in auxin, CK, and ABA MST pathway, respectively. *VvYUC10–4*, *VvTAR4–1*, *VvAUX*, *VvARF2–2*, and *VvSAUR2* of auxin MST pathway, *VvNCED1*and *VvPP2C49* of ABA MST pathway, and *VvCKX5* of CK MST pathway exhibited higher expression levels than other members of their corresponding gene families (Fig. 4; Additional file 2: Table S2).

#### Auxin MST pathway

Both *VvYUC10–4* and *VvTAR4–1* key enzymes in endogenous auxin biosynthesis showed the increased expression peak at GBS and dramatically decreased at BVS. *VvARF2–2*, which is a transcriptional regulator repressing a series of auxin response, exhibited the highest expression gene of 18 ARF gene members. Most of them displayed the highest expression at GBS and then dramatically decreased until BVS of berries. Similar to *VvARFs*, AUX/IAA protein was also abundant at GBS. Another important element in auxin MST pathway is *VvSAUR*, and its transcription is dependent on the level of active auxin. Different from *VvARFs* and AUX/IAA proteins, *VvSAUR2* showed high transcription levels through berry development process.

#### ABA MST pathway

*VvNCED1* and *VvNECD2–1* genes, which are rate-limiting enzymes for ABA biosynthesis, are highly expressed at GBS. In addition, seven *VvCYP707As* were identified in this work for ABA metabolism, and five of them were abundant at GBS. Five ABA receptors were found, but only *VvPYL9* was highly expressed in grape berry and primarily at GBS. Then, 51 *VvPP2C* gene members were identified from our database. *VvPP2C25–1* and *VvPP2C49* were highly expressed than others, and 37 members of PP2C gene family expressed at GBS as well. *VvABI5s* is primarily expressed at GBS and is higher than other stages during SBD.

#### CK MST pathway

In CK MST pathway, compared with the other six members of CKX family, *VvCKX5* displayed the highest expression levels in berry. A similar trend for*VvCKX3–2*, *VvCKX5*, and *VvCKX7* was detected at GBS and then dramatically decreased until BRS. Only *VvLOG1–2* showed highest levels among the 10 LOG members at GBS. *VvCKXs* was highly expressed than *VvLOGs* at GBS, so we presume that cytokinin may primarily be accumulated during SHS, BVS, and BRS. These results are inconsistent with the variation of endogenous CK content during SBD (Fig. 1d).

### Organ-specific expression pattern analysis of genes in hormone MST pathway at the global transcriptome level of grape berries

**Fig. 5:**
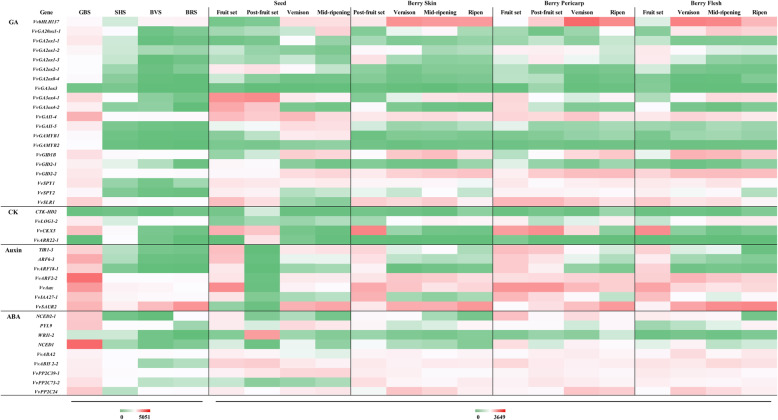
Validation of temporal and spatial expression patterns of genes involved in hormones MST pathway. Data from global transcriptomic at four developmental stages of seed (Veraison, Mid-ripening, Fruit set, Post-fruit set), berry skin (Post-fruit set, Veraison, Mid-ripening, Ripeing), berry pericarp (Fruit set, Post-fruit set, Veraison, Ripening) and berry flesh (Post-fruit set, Veraison, Mid-ripening, Ripening) compared with expression at four stages (GBS, SHS, BVS and BRS) from RNA-seq database

To evaluate the validity of our sequencing results, based on the data from *V. vinifera* cv. Corvina global gene expression atlas from the GEO DataSets (GSE36128), which comprising 54 grape tissues, organs or developmental stages [9]. The 64 gene tissue-specific expression patterns during seed, berry skin, berry pericarp, and berry flesh development were identified in this work (Fig. 5; Additional file 3: Table S3). Figure 5 shows that 8, 1, 3, and 2 gene expression patterns in seed, berry skin, pericarp, and flesh were similar to our data, respectively. Additionally, 50 genes displayed the same trend in at least two tissues from our database, indicating that multitissues regulated these gene expression. We found genes expressed differently in the same period of different tissues due to their function in various developmental process. For example, *VvGA20ox1–1* and *VvGA3ox4–1* were detected low in veraison and ripe berry in our data, whereas both were highly expressed at veraison and ripe period in berry skin and flesh from the GEO DataSets. It supposed that accumulation of anthocyanin and glucose and fructose are relative to GA, thus two genes highly expressed for GA biosynthesis at two periods.

### Data analysis of GA MST pathway genes between RT-qPCR and Illumina RNA-seq

**Fig. 6:**
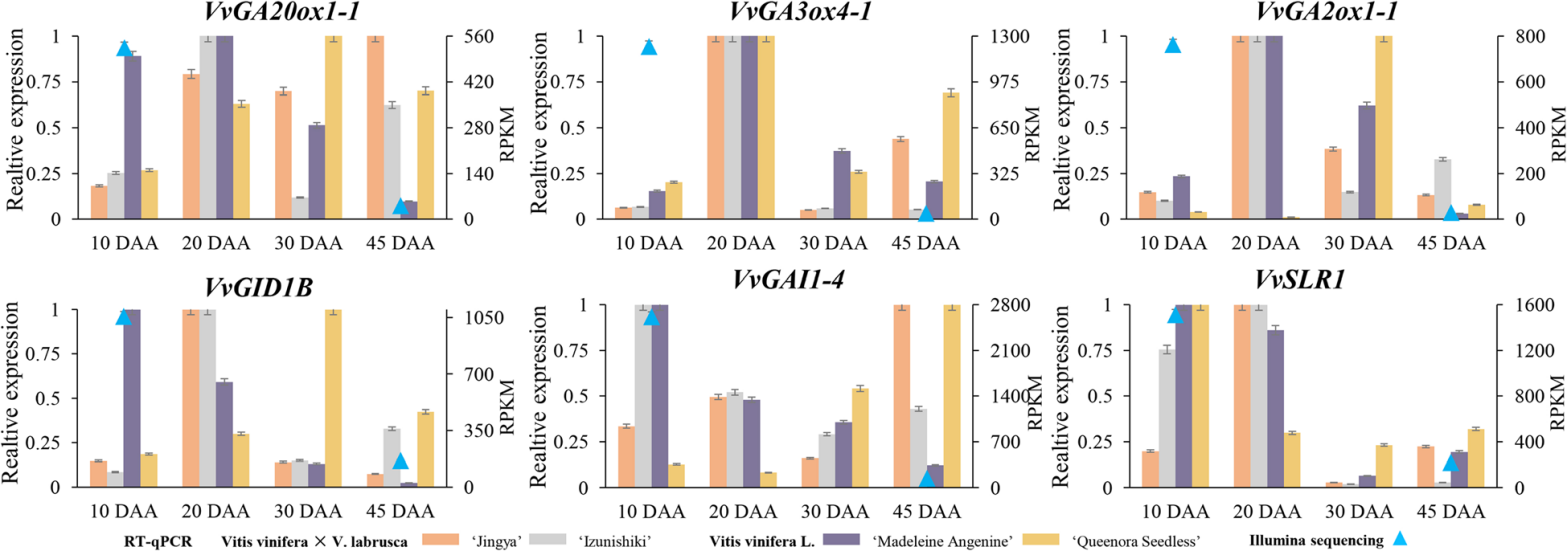
Data analysis of GA MST pathway genes between RT-qPCR (10 DAA, 20 DAA, 30 DAA and 50 DAA) and Illumina RNA-seq (10 DAF and 40 DAF) in grape berry

To validate expression patterns obtained from RNA-Seq, six genes (i.e., *VvGA20ox1–1*, *VvGA3ox4–1*, *VvGA2ox1–1*, *VvGID1B*, *VvGAI1–4*, and *VvSLR1*) that were the core genes in GA MST pathway and highly expressed in green berry were selected to validate by using RT-qPCR analysis (Fig. 6). The expression profiles of RT-qPCR were a similar tendency with RNA-Seq results, indicating that the data from RNA-Seq could represent genes relative expression levels at different development stage in grape berry. The relative expression levels of *VvGA20ox1–1*, *VvGA3ox4–1*, *VvGA2ox1–1*, *VvGID1B*, *VvGAI1–4*, and *VvSLR1* were highly detected at GBS and dramatically decreased at SHS from Illumina sequencing. Data exhibited that the six genes showed the highest expression at 10 days after anthesis (DAA) or 20 DAA in RT-qPCR results, which were approximately consistent with Illumina sequencing. This difference may be caused by different species with slightly different time points during the development process.

## Discussion

According to previous reports, GA regulatory networks are involved in organogenesis and development of fruit in tomato [10] and strawberry [11]. Moreover, genes involved in GA MST pathway are associated with fruit development in pepper [12], pea [13], tomato [14], and *Arabidopsis* [15]. Few studies [16] have been conducted on the GA MST and GA-mediated multihormone MST pathway mechanisms in grape. Hitherto, no reports are available on the identification and comparative profiling of GA mediated pathway and multihormones like auxin, ABA, and CK mediated pathway genes during SBD in grape.

### Genes in GA MST pathway during SBD

Gene duplication events are defined as either tandem or segmental duplication, which have been identified as two of the main causes for genomic rearrangements and expansions [17]. Holub [18] reported that a tandem duplication event involves three or more copies in chromosomal regions of less than 200 kb. Three tandem duplication event regions on Chr4, 9, and 19 were found in our present study. Moreover, three segregation duplication events were also identified, indicating that some GA biosynthesis genes (i.e., *VvGA3oxs* and *VvGA20oxs*) were possibly generated by gene duplication. Overall, the collinearity events can also support the GA biosynthesis gene evolutionary relationship and functional prediction. Based on the phylogenetic tree, we found that the more similar the functions of genes are, the closer they are in relationship. The closer evolutionary relationship may explain the highly conserved motif and large variation in the structures of genes in GA MST pathway, indicating that the grape genome changed significantly during its extensive evolutionary processes.

*VvGA3oxs* and *VvGA20oxs* are particularly important in controlling bioactive GA levels [19], and *VvGA2oxs* modulate the endogenous GA balance by GA inactivation. In the present study, *VvGA20oxs*, *VvGA3oxs* and *VvGA2oxs* were multicopy genes and exhibited temporal-specific expression pattern in berries, which agree with the studies on rice [20] and *Arabidopsis* [21]. Field observations and few physiological studies [22] point out that grape SBD are strictly correlated, and seeds are generally a metabolic center of phytohormones [23]. From our studies, grape seeds are one of the organs for endogenous GA biosynthesis, whereas GBS and SHS are important phases for grape SBD. Expression analysis of GA genes indicated that *VvGA20ox1–1*and *VvGA3ox4–1* may be key factors in endogenous GA biosynthesis during SBD, especially at GBS. Combined with high expression level of *VvGA2ox1–1*at GBS, it may be the key factor in modulating the endogenous GA balance by GA inactivation at GBS. Simultaneously, GBS and SHS are the crucial phases for endogenous GA metabolism process, consistent with endogenous GA contents, which increase from GBS to SHS in berries and reach the expression peak at SHS. This phenomenon may be derived from the fact that seeds are the important sites for endogenous GA biosynthesis, whereas the period from GBS to SHS is significant for the development of seeds.

DELLA proteins, which are GA signal pathway receptors, are the core acting elements of GA MST pathway [24]. Our study showed that GBS is a key stage of responding to GA and GA signal transduction during SBD. *VvGAI1–4* and *VvSLR1* may also be crucial core acting factors of GA signal transduction at GBS. GID1B, which is a GA receptor, plays a key role in switching on GA signal transduction. Given that *VvGID1B* exhibits high expression at GBS, GBS may be the active stage for strong response to GA signal. Although previous evidence showed that two putative GA receptors increased during grape berry development [25], this discrepancy may be caused by different grape varieties used in the two studies. *VvGID2* is a homology with rice F-box protein GID2 [19], which may be required for GA-induced degradation of DELLA via the proteasome pathway. In the present work, *VvGID2–1* and *VvGID2–2* may play important roles at GBS, and *VvGID2–2* may also function at BRS. GBS and SHS are significant phases for GA biosynthesis, inactivation, and signal transduction of grape berry.

### Regulator genes in GA MST pathway during SBD

GA signal can be positively or negatively modulated by many regulators like transcription factors and other regulatory genes. *VvGAMYB* [26], *VvSHI* [27], and *VvPKL* [28], which act downstream of DELLA, are identified as positively acting components in *Arabidopsis*. *VvGAMYB*, *VvSHI*, and *VvPKL* were expressed at low levels throughout the four stages in the present study. *VvSHI* and *VvPKL* play important roles in flowering regulatory network, leaf development, or repressing expression of embryonic traits but not in seed and fruit development [27, 28], whereas *VvGAMYB* is involved in GA–DELLA (*VvSLR1*)–VvmiR159c–*VvGAMYB* signal pathway, which modulate grapevine floral development and GA-induced grapevine parthenocarpy [2]. Similar to *AtSPY* [29], *VvSPY2* acts as GA signaling repressor in grape berry as well. Additionally, *VvbHLH137* is a DELLA direct target [24] and possesses high expression levels in berries as well, indicating that *VvbHLH137* may act as a crucial DELLA downstream target gene involved in SBD. Our results showed that *VvSPY1*, *VvSPY2*, and *VvbHLH137* may be crucial regulatory factors in modulation of GA signal transduction during grape SBD.

Additionally, many GA-related miRNAs and their targets have been identified in orange [30] and *Oryza sativa* [1], which can facilitate the exploration of miRNA roles in GA MST pathway in grape berries. A functional interaction between miR159a and its target *GAMYB* in fruit development has been demonstrated in grape [29] and tomato [31]. Moreover, an increasing number of studies confirmed that miR319b [32], miR477 [33], and miR396 [34] are fruit development-related miRNAs, and miR159, miR319, miR477, and miR396 that responded to GA are also proven [35]. Overall, VvmiR159, VvmiR319, VvmiR477, and VvmiR396 may be induced by GA and involved in SBD. Therefore, transcription factor and miRNAs may contribute to maintain GA homoeostasis between GA synthesis and signal transduction process.

### Genes in multihormone MST pathways during SBD

Auxin acts as a positive regulator during fruit set and growth and negative regulator during fruit maturation and ripening [36]. In the present study, IAA content was highest at SHS and decreased until BRS. GBS was a key phase for *VvYUC10–4*and *VvTAR4–1* involved in auxin biosynthesis, with auxin accumulation until SHS possibly reaching the highest level. Auxin produced by the seed in growing fruit acts to prevent ripening and premature dispersal before the seeds are fully developed [37]. Similarly, IAA content of berries decreased from SHS when seeds were fully developed. Furthermore, GBS was also an important phase for genes expressing during auxin signal transduction, such as *VvARF2–2* and AUX/IAA protein. In tomato, auxin indirectly regulates ARF activity by promoting turnover of Aux/IAA proteins [38]. This promotion allows the ARFs to become active and impose their regulatory influence on the expression levels of auxin-responsive genes [38]. Thus, similar to tomato [39, 40], an ARF–IAA may interact to mediate auxin signaling and be directly involved in grape SBD. We indicated that *VvSAUR* may be associated with a new function in stimulating grape berry ripening. *VvSAUR2* especially showed high expression during BVS and BRS berry, which was different from *ZmSAUR2* involving in auxin-mediated cell elongation in maize [41]. A rice *OsSAUR39* gene negatively regulates auxin synthesis and transport [42]. *VvSAUR2* was similar to *OsSAUR9*, whose expression pattern was opposite to active auxin level in berry. This phenomenon may be caused by a complex network for *VvSAURs* responding auxin in grape.

In previous study [43], ABA can be considered as a candidate ripening control factor in grape, but we considered that ABA may also be involved in regulating grape seed development. ABA is important during fruit development [44], ripening [45], and seed development [46]. Traditionally, *VvNCED1*genes are expressed only at the beginning of ripening when ABA accumulation is high and ABA content is low in unripe fruit, increasing during fruit ripening [43]. Nevertheless, in the present study, a contradictory conclusion was drawn that ABA levels were much higher in green berries than in ripening berries (Fig. 1d). Similar to apple [44], ABA content also decreases from young fruit to older fruit and then increases during the ripening process in apple fruits. The highest ABA content in green berries was detected, implying that ABA may function as an indispensable hormone regulating seed development. Furthermore, *VvNCEDs* were noted to be at a higher level at GBS. *NCED* gene encodes a key rate-limiting enzyme in ABA biosynthesis [43], and CYP707A is a key enzyme for ABA metabolism. Both were abundantly detected at GBS, but *VvNCEDs* expression was significantly higher than *VvCYP707As* expression, suggesting that *VvNCEDs* played a stronger role than *VvCYP707s* at GBS. The only reasonable explanation is that ABA levels required for seed development are higher than those required for ripening process in grape. In *Arabidopsis* [47, 48], two peaks of ABA accumulation appear in the mid and late stages during seed development, but only one peak of ABA level at GBS was observed in this study. This result may be caused by sampling times or different species. Furthermore, *VvPP2C25–1* and *VvPP2C49* may be the key factors of PP2C family in negatively regulating endogenous ABA content and response during grape SBD, especially at GBS.

Generally, cytokinin activities were found to peak shortly in young fruit progressing through the cell division phase, whereas activities were low or undetectable in ripening fruit. However, recent studies report of greatly increased cytokinin in the flesh of ripening kiwi fruit [49] and grapes [50], suggesting that cytokinin is also implicated in the control of ripening-related processes. In the present study, ZR levels reached two peaks at SHS, being active during cell division and being ripening phase at BRS. In grape [51], genes that regulated cytokinin biosynthesis (IPTs), activation (LOGs), and degradation (CKXs) are found to be expressed in four stages of berry development. Previous studies showed that *VvIPTs* are also found in other grape organs [51], in agreement with reports from *Arabidopsis* [52], tomato [53], and soybean [54]. The irreversible degradation of cytokinins by CKXs enzymes in grape berries is restricted to early developmental stages [24, 55]. Additionally, as previously reported [24, 55], *VvCKX5* shows the highest expression at GBS and then exhibits progressive decrease of *VvCKX5* transcripts, which may contribute to the large increase in isopentenyladenine concentrations in post-veraison of grape berries [56, 57].

### Model of GA-mediated multihormone regulatory networks of SBD in grape

**Fig. 7:**
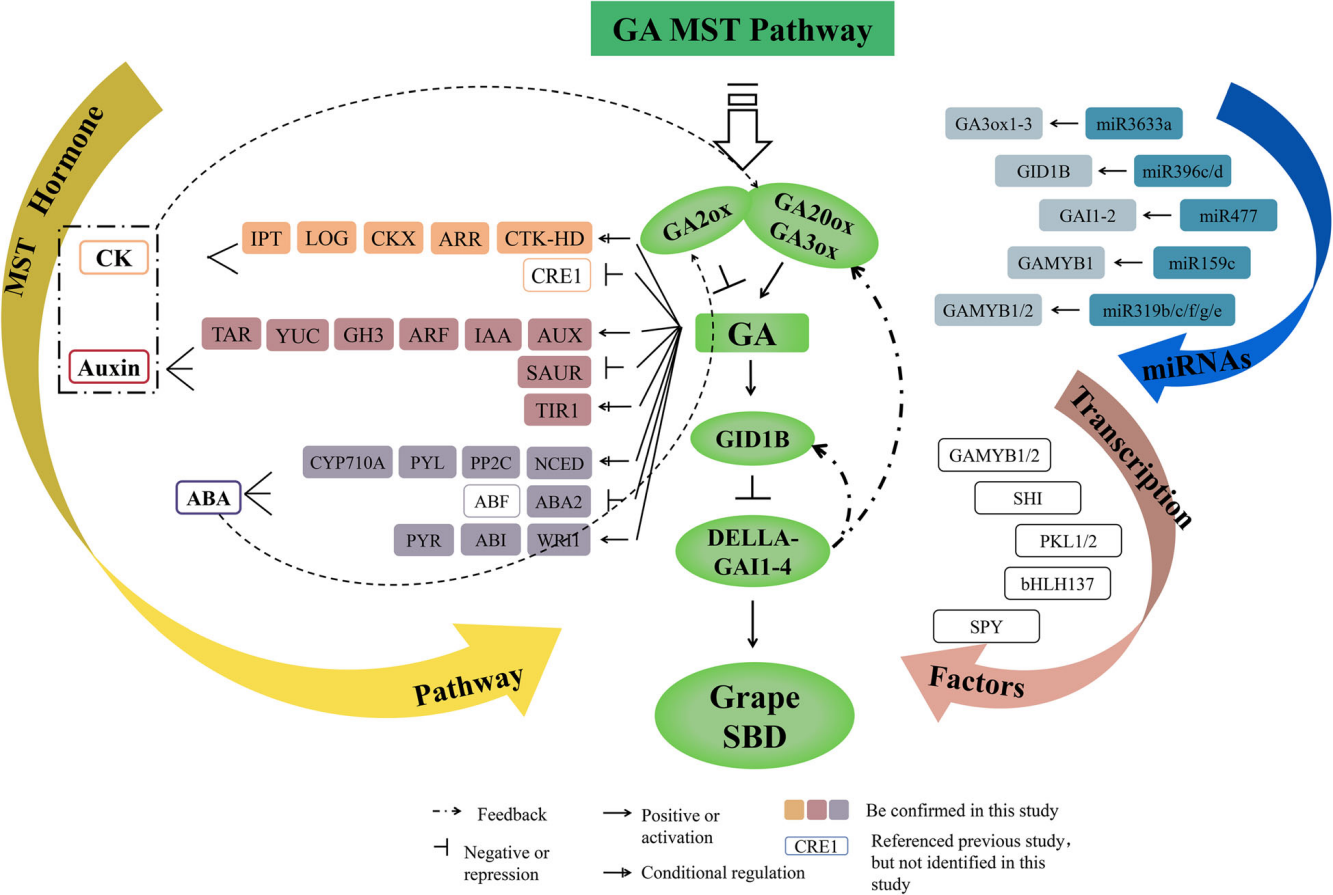
Schematic representation of plant hormone metabolism and signaling-pathway gene network during SBD

Many studies related to one or two hormones regulating berry development have recently been reported [1, 53, 58, 59]. Nevertheless, few pertinent evidence are available on hormonal crosstalk during berry development in grape. A complex berry development regulatory network of GA downstream plant hormone signaling, and cross talk were demonstrated in this study to clarify the key nodes and nexuses in grape berries (Fig. 7). In the present study, biosynthesis enzymes *GA20oxs* and*GA3oxs* and GA receptor *GID1B* were highly expressed at GBS, meanwhile high expression levels of repressor DELLA proteins of GA MST pathway was also detected at this stage. Furthermore, *GA3ox1*, *GA20ox2*, and *GID1B*may be direct DELLA targets in *Arabidopsis* [24]. Thus, we supposed that a feedback mechanism existed as *VvGAI1–4*-*VvGA20ox*, *VvGAI1–4*-*VvGA3ox*, and *VvGAI1–4*-*VvGID1B* to maintain the balance of GA content in regulating grape berry development. Furthermore, other hormones [60, 61] influence GA levels by regulating *VvGA20oxs* and *VvGA3oxs* expression levels. Overall, GA20oxs, GA3oxs, and GA2oxs are not merely core acting enzymes in GA metabolism, but upstream factors also respond to other hormones in GA MST pathway (Fig. 7). In addition, the crosstalk between GA and IAA [62], ABA [63], CK [61] in regulating SBD is also proved [63]. Many studies have demonstrated that bioactive GA controls plant growth and development, at least in part, by depressing the repressive effect of DELLA proteins [64]. Additionally, interactions as GA–auxin [58] and GA–CK [65] hormone signal are also mediated by DELLA proteins to control various processes in plant. GA may act downstream of auxin involved in early fruit development of *Arabidopsis* [66] and tomato [36]. Additionally, ABA also regulates tomato fruit set along with GA [66]. Different from the active ABA accumulation in the mid and late stages during seed development [43], GA biosynthesis appears to be more active in the early stage of seed development [67]. Thus, further functional studies are needed to validate the networks of GA and multihormone in grape SBD. This regulatory network could facilitate dissecting the molecular mechanisms underlying genes of other hormones MST pathway, respond to GA, and provide insights into the relationship between hormones and SBD in grape.

## Conclusion

In the present study, total endogenous GA_3_ content significantly decreased during SBD, and a total of 48 related genes in GA M (31) and ST (17) pathway were identified by using transcriptome sequencing. Most genes of *VvGA20ox*, *VvGA2ox*, and *VvGA3ox* family in GA M pathway expressed variously during SBD, yielding a metabolism flux toward GA biosynthesis. In GA M pathway, out of 31 genes, *VvGA20ox1–1*, *VvGA3ox4–1*, and *VvGA2ox1–1* may be the major factors at GBS in interacting GA accumulation rate. Furthermore, *VvGA20ox1–1* and *VvGA3ox4–1* were more active than *VvGA2ox1–1*, being consistent with the fact that GA was synthesized for seed development. The visible correlation between endogenous GA_3_ content and gene expression profiles suggested that transcriptional regulation of GA biosynthesis pathway genes is a key mechanism of GA accumulation at the SHS. We revealed feedback regulation as *VvGA3oxs-VvGAI1–4*, *VvGA2oxs-VvGAI1–4*, and *VvGID1B-VvGAI1–4*, maintaining the balance of GA_3_ content in berries. Moreover, 11 miRNAs may be involved in modulation of GA MST pathway by mediating their target genes, such as *VvGA3ox*, *VvGID1B*, and *VvGAMYB*. Moreover, we found that crosstalk between GA and auxin, ABA and CK may modulate the complex process during SBD by the interaction network of genes of multihormone pathway. Lastly, based on the expression characterization of multihormone MST pathway genes, a putative schematic model of GA-mediated multihormone regulatory network during SBD was proposed, which may provide novel insight to explore GA-mediated regulatory networks during SBD in grape. Our results may provide novel insight to explore GA-mediated regulatory networks during SBD in grape.

## Methods

### Plant material

Three-year-old ‘Fujiminori’ grape trees grown in Nanjing, China (N32°02′12.77′′, E118°37′33.25′′) were chosen as the experimental material. The plant materials were grown under common field conditions at the Jiangsu Vocational College of Agriculture and Forestry grape farm, Jurong, China (our long-term partners). Berry samples were collected at different stages: green berry stage (GBS) [10 days after flowering (10 DAF)], stone-hardening stage (SHS) (40 DAF), berry veraison stage (BVS) (65 DAF) and berry ripening stage (BRS) (90 DAF) throughout the growing season. A total of 12 samples, comprising four stages berry, and including three biological replicates, were sequenced. RNA for Illumina sequencing was purified from 40 uniform and average-sized berries with seeds sampled from 20 clusters to study a representative biological selection of transcripts at each stage.

### Total RNA extraction, construction of cDNA library, and Illumina deep sequencing

Total RNA of different stages (i.e., GBS, SHS, BVS, and BRS) berry samples was extracted using TRIzol reagent (Invitrogen, Carlsbad, CA, USA). Sequencing libraries were generated using NEBNext® UltraTM RNA Library Prep Kit for Illumina® (NEB, USA) following manufacturer’s recommendations. The transcriptome were sequenced using Illumina HiSeq™ 2500 Shanghai Hanyu Biotechnology Company (Shanghai, China) [68].

### Accession code

The RNA-seq data have been deposited into the NCBI under the accession number SRP068911 (https://www.ncbi.nlm.nih.gov/sra?term=SRP068911) and GSE153169 (https://www.ncbi.nlm.nih.gov/geo/query/acc.cgi?acc=GSE153169).

### Identification and analysis of the GA MST and multihormone MST pathway genes

GA MST pathway and multihormone MST pathway genes were identified via BLAST P search from NCBI using *Arabidopsis* gene sequences as queries. We gained the grape genome and a set of annotated gene sequences from Grape genome data Get Data: Grapevine V1 Annotation. And a survey was conducted to affirm these genes by functional annotation from grape transcriptome and confirm the reliability of the initial results (RNA-Seq data accession SRP068911) [69].

### Morphological, physiological, and biochemical variations during SBD

The gene location in the chromosome of each GA MST pathway genes was obtained from the grape genome (http://www.genoscope.cns.fr/externe/ Genome Browser/Vitis/). As Leng [69] described, gene duplication events was detected using MCScanX software (http://chibba.pgml.uga.edu/mcscan2/). We used protein sequences to construct phylogenetic trees in MEGA 6.0 software using the neighbor-joining method with 1000 bootstrap replicates [70], and intron/exon structures were predicted using the Gene Structure Display Server (http://gsds.cbi.pku.edu.cn/index.php), and conserved domains were searched using SMART (http://smart.embl-heidelberg.de/). Motifs were searched using the MEME 5.05 online program (http://meme-suite.org/tools/meme). The optimized parameters of MEME were employed as follows: number of repetitions, any; maximum number of motifs, 15; and the optimum width of each motif, between 6 and 50 residues.

### Examination of endogenous GA_3_, IAA, ZR, and ABA

Berries of four stages, G, SH, V, and R, were chosen. Endogenous GA_3_, IAA, ZR, and ABA levels were determined by using an enzyme-linked immunosorbent assay kit (ELISA) (Rapidbio, USA), according to the manufacturer’s instructions and as described in previous study [71]. Each berry with seed was sampled from 40 berries of 20 clusters as replicates.

### Expression analysis of genes of GA and multihormone MST pathway

Differential expression was analyzed and calculated using the edgeR R packkage [72]. If transcripts with “FDR < 0.001” and “|log2 fold-change (|log2FC|) ≥ 1”, they will be identified as significant differences in transcription level. And transcripts with |log2FC| < 0.25 were supposed to have no change, while transcripts with 0.25 < |log2FC| < 1 were considered as “changed slightly” in expression level [69].

### RT-qPCR validation in different grape varieties

RT-qPCR was used to verify the expression patterns of GA MST pathway genes from RNA-Seq. Total RNA samples of four stages (i.e., 10, 20, 30, and 50 DAA) of *Vitis vinifera* × *V. labrusca* (‘Jingya’ and ‘Izunishiki’) and *V. vinifera*l. (‘Queenora Seedless’ and ‘Madeleine Angenine’) were extracted using our modified cetyltrimethyl ammonium bromide (CTAB) method [73]. Purified RNA samples were reverse transcribed using the PrimeScript RT Reagent Kit with gDNA Eraser (Takara, Dalian, China). Gene specific primers were designed using Primer3 software (http://primer3.ut.ee/) (Additional file 4: Table S4). For each reaction, a total volume of 20 μl contained 10 μl of 2 × SYBR green reaction mix, 2.0 μl of diluted cDNA, and 0.2 μM of each primer. And three replicates of each cDNA sample were performed and normalized according to the internal control. The relative gene expression data were analyzed using the 2^−ΔΔCT^ method [74].

## Supplementary information

**Additional file 1: Table S1.** The gene expression patterns of GA MST pathway genes in four stages of grape SBD.

**Additional file 2: Table S2.** The gene expression patterns of auxin, ABA and CK MST pathway genes in three stages of grapevine SBD.

**Additional file 3: Table S3.** 64 genes expression patterns of GA, auxin, ABA and CK MST pathway in seed, berry skin, pericarp and flesh from a global transcriptomic atlas.

**Additional file 4: Table S4.** The primers sequences of GA MST pathway genes for qRT-PCR.

**Additional file 5: Table S5.** The name, stage and type of data used in this study.

## Data Availability

The RNA-seq data have been deposited into the NCBI under the accession number SRP068911 (https://www.ncbi.nlm.nih.gov/sra?term=SRP068911) and GSE153169 (https://www.ncbi.nlm.nih.gov/geo/query/acc.cgi?acc=GSE153169). All data generated or analyzed during this study are included in this published article and its additional files.

## Abbreviations

ABA: Abscisic acid
CK: Cytokinin
AUX/IAA: Auxin–response repressor protein/Indole Acetic Acid
ARF: Auxin response factor
CKX: Cytokinin dehydrogenase
GA2ox: Gibberellin 2-oxidase
GA20ox: Gibberellin 20-oxidase
GA3ox: Gibberellin 3-oxidase
GID1: Gibberellin insensitive dwarf1
NCED: 9-cis-epoxycarotenoid dioxygenase
PKL: PICKLE
PP2C: Protein phosphatase 2C
SAUR: Small auxin-upregualted RNAs
SHI: Short internodes
SPY: Spindly
TAR: Tryptophan aminotransferase
YUC: Yucca
