# Supplementary material for: Genome-wide identification and characterization of gibberellin metabolic and signal transduction (GA MST) pathway mediating seed and berry development (SBD) in grape (Vitis vinifera L.)
Source: BMC Plant Biol. 2020 Aug 21;20:384. doi: 10.1186/s12870-020-02591-1 (PMC7441673; doi:10.1186/s12870-020-02591-1)
Supplement: Supplementary file 1 — Additional file 1: Table S1. The gene expression patterns of GA MST pathway genes in four stages of grape SBD. [file 12870_2020_2591_MOESM1_ESM.pdf]

Additional file 1:Table S1 The gene expression patterns of GA MST pathway genes in four stages of grape SBD.

GA metabolic pathway

#Gene	Abbreviation	Length	Function	Green-RKPM	Stone hardening-RPKM	Veraison-RPKM	Ripe-RPKM	Veraison/Stone hardening Log2	Ripe/veraison log2	Stone hardening/Green log2	Ripe/Green log2
VIT_215s0046g02550	*VvGA20ox1-B1*	987	gibberellin 20 oxidase 1-B-like	1.061963108	0.027319153	0.023253818	0.024501819	-0.232445147	0.075421226	-5.2806770768212	-5.43770099799371
VIT_216s0050g00640	*VvGA20ox1-B2*	1178	gibberellin 20 oxidase 1-B-like	0.023	0.045779294	0.019483462	0.041058226	-1.232445147	1.075421226	0.993061351149903	0.836037429977386
VIT_203s0063g01150	*VvGA20ox1-1*	1292	gibberellin 20 oxidase 1-like	525.3658923	41.03036542	3.766038631	7.599394578	-3.445572299	1.012836689	-3.67855863289385	-6.11129424322177
VIT_202s0234g00010	*VvGA20ox1-2*	1074	gibberellin 20 oxidase 1-like	0.023	0.025106149	0.021370129	0.022517035	-0.232445147	0.075421226	0.126406896994952	-0.0306170241775667
VIT_209s0002g05290	*VvGA20ox1-3*	963	gibberellin 20 oxidase 1-like	4.233074983	0.112000017	0.023833352	0.100449824	-2.232445147	2.075421226	-5.24013518576528	-5.3971591069378
VIT_218s0001g01390	*VvGA20ox2-1*	1264	gibberellin 20 oxidase 2-like	0.023	0.021332282	0.018157847	0.019132354	-0.232445147	0.075421226	-0.10859557319674	-0.26561949436926
VIT_215s0048g01320	*VvGA20ox2-2*	1185	gibberellin 20 oxidase 2-like	8.461299426	0.022754434	0.01936837	0.040815688	-0.232445147	1.075421226	-8.53858773654743	-7.69561165771995
VIT_204s0044g01520	*VvGA20ox2-3*	1252	gibberellin 20 oxidase 2-like	7.404239581	0.021536745	0.018331884	0.019315731	-0.232445147	0.075421226	-8.42540757826939	-8.5824314994419
VIT_204s0044g01650	*VvGA20ox2-4*	1217	gibberellin 20 oxidase 2-like	9.518360476	1.28505525	0.188590949	0.019871237	-2.768498048	-3.246506869	-2.88888270391234	-8.90388762021243
VIT_209s0002g05280	*VvGA20ox3-1*	1236	gibberellin 20 oxidase 3-like	5.290126097	0.56720397	0.01856919	0.019565773	-4.932884865	0.075421226	-3.22136257475204	-8.07882621406565
VIT_209s0002g05320	*VvGA20ox3-2*	942	gibberellin 20 oxidase 3-like	81.39874646	0.028624208	0.024364669	0.025672288	-0.232445147	0.075421226	-11.4735550753701	-11.6305789965427
VIT_216s0022g02310	*VvGA20ox*	1321	gibberellin 20-oxidase	9.518360476	0.122470874	0.034748703	0.036613619	-1.817407648	0.075421226	-6.28020249685185	-8.02218891874553
VIT_209s0002g05340	*VvGA3ox1-1*	1286	gibberellin 3-beta-dioxygenase 1-like	0.023	0.020967344	0.017847215	0.018805051	-0.232445147	0.075421226	-0.133489752347587	-0.290513673520099
VIT_204s0008g04940	*VvGA3ox1-2*	1174	gibberellin 3-beta-dioxygenase 1-like	2.118998898	0.091870542	0.039099691	0.082396235	-1.232445147	1.075421226	-4.52763668385035	-4.68466060502287
VIT_209s0002g05300	*VvGA3ox1-3*	1190	gibberellin 3-beta-dioxygenase 1-like	2.118998898	0.022658827	0.01928699	0.020322097	-0.232445147	0.075421226	-6.5471658489356	-6.70418977010812
VIT_209s0002g05270	*VvGA3ox1-4*	1297	gibberellin 3-beta-dioxygenase 1-like	193.4475997	0.020789517	0.017695851	0.074582252	-0.232445147	2.075421226	-13.1837989357548	-11.3408228569273
VIT_209s0002g05350	*VvGA3ox3*	1022	gibberellin 3-beta-dioxygenase 3-like	4.233074983	6.279288634	0.022457454	0.023662715	-8.127262911	0.075421226	0.568895084448501	-7.48294660003196
VIT_204s0008g04920	*VvGA3ox4-1*	1299	gibberellin 3-beta-dioxygenase 4-like	1227.2568	97.39423202	11.16655863	5.287186911	-3.124651697	-1.078612403	-3.65545701603598	-7.85872111573233
VIT_204s0044g02010	*VvGA3ox4-2*	929	gibberellin 3-beta-dioxygenase 4-like	527.4800215	11.02940966	11.90810753	0.026031534	0.110588581	-8.83746811	-5.57968897380796	-14.3065685033114

VIT_202s0025g03440	*VvGA3ox4-3*	1199	gibberellin 3-beta-dioxygenase 4-like	10.57542238	0.179909953	0.019142217	0.020169554	-3.232445147	0.075421226	-5.87729647708426	-9.03432039825678
VIT_219s0140g00120	*VvGA2ox1-1*	1223	gibberellin 2-beta-dioxygenase 1-like	763.205432	29.05850977	1.613925255	3.519727327	-4.170315048	1.124889902	-4.71503881637343	-7.76046396158761
VIT_213s0067g01150	*VvGA2ox1-2*	1296	gibberellin 2-beta-dioxygenase 1-like	293.8687397	28.62844883	18.06369508	15.26383901	-0.664356465	-0.242975178	-3.35965049196654	-4.2669821348994
VIT_219s0140g00140	*VvGA2ox1-3*	1138	gibberellin 2-beta-dioxygenase 1-like	106.7682986	26.7744505	1.210097634	3.825125756	-4.467661609	1.660383727	-1.99555447757746	-4.80283235972286
VIT_205s0077g00520	*VvGA2ox2-1*	1187	gibberellin 2-beta-dioxygenase 2-like	82.45581115	15.94669832	2.474974189	8.434611756	-2.687772368	1.768908184	-2.3703634203204	-3.28922760429816
VIT_207s0005g01920	*VvGA2ox2-2*	1008	gibberellin 2-beta-dioxygenase 2-like	0.023	0.026750004	0.022769364	0.023991364	-0.232445147	0.075421226	0.217905251480445	0.0608813303079285
VIT_209s0002g05310	*VvGA2ox2-3*	813	gibberellin 2-beta-dioxygenase 2-like	0.023	0.033166057	0.02823065	0.02974575	-0.232445147	0.075421226	0.528073632905336	0.371049711732818
VIT_201s0010g01650	*VvGA2ox8-1*	1092	gibberellin 2-beta-dioxygenase 8-like	6.347181498	0.098769246	0.021017874	0.088583498	-2.232445147	2.075421226	-6.00591039179329	-6.16293431296581
VIT_219s0177g00030	*VvGA2ox8-2*	1287	gibberellin 2-beta-dioxygenase 8-like	10.57542238	2.514126259	1.426667814	2.029367418	-0.817407648	0.508380633	-2.07258627628222	-2.38161329089979
VIT_210s0116g00410	*VvGA2ox8-3*	1628	gibberellin 2-beta-dioxygenase 8-like	155.3932727	0.132501249	0.056391937	2.198481369	-1.232445147	5.284874592	-10.1957023752004	-6.14327293074397
VIT_206s0004g06790	*VvGA2ox8-4*	1197	gibberellin 2-beta-dioxygenase 8-like	156.4503373	10.54231741	1.112103651	0.020203254	-3.244828872	-5.782559769	-3.89144082986593	-12.9188294706219
VIT_210s0003g03490	*VvGA2ox*	1191	gibberellin 2-beta-dioxygenase-like	132.1378504	5.071315639	1.271872559	0.324880538	-1.99540595	-1.968972893	-4.70353989977196	-8.66791874300208

GA signal transduction pathway

#Gene	Abbreviation	Length	Function	Green-RKPM	Stone hardening -RPKM	Veraison-RPKM	Ripe-RPKM	Veraison/Stone hardening Log2	Ripe/veraison log2	Stone hardening/Green log2	Ripe/Green log2
VIT_213s0067g01630	*VvGAMYB1*	2516	transcription factor GAMYB-like	151.1650141	3.408010061	1.550778274	1.614782818	-0.232445147	0.075421226	-5.4710508731757	-6.54864032685382
VIT_206s0009g02480	*VvGAMYB2*	1932	transcription factor GAMYB-like	73.99929366	2.12139163	1.164207458	0.275379136	-0.232445147	0.075421226	-5.12442861376515	-8.06994842974396
VIT_203s0038g00310	*VvSHI*	1274	Lateral Root Primordium type 1 C-terminal; Protein of unknown function DUF702; Zinc finger lateral root primordium type 1;	1.061963108	0.259269271	0.03678128	0.038755281	-0.647482647	-4.094503775	-2.03421052050589	-4.77619694239957
VIT_204s0008g05880	*VvPKL*	4688	CHD3-type chromatin-remodeling factor PICKLE-like	699.7815547	2.749316121	0.274164896	0.588075008	0.157592679	0.201739941	-7.99168802750468	-10.2166887424932
VIT_208s0007g02910	*VvSPY1*	3344	probable UDP-N-acetylglucosamine--peptide	903.7950264	14.06256675	5.257435148	16.8502026	-2.817407648	0.075421226	-6.00606376854161	-5.74515777494942

			N-acetylglucosaminyltransferase SPINDLY-like								
VIT_217s0000g09890	*VvSPY2*	2074	probable UDP-N-acetylglucosamine--peptide N-acetylglucosaminyltransferase SPINDLY-like	265.3279947	8.268614575	2.92150476	2.448646076	-1.364741744	-0.595017111	-5.00398746915432	-6.75964882794507
VIT_217s0000g00430	*VvbHLH137*	1564	transcription factor bHLH137-like	189.2193412	29.75695085	361.1782431	400.6015399	-0.974376994	-0.290706673	-2.66876095374283	1.08210840464936
VIT_207s0129g01000	*VvGID2-1*	777	F-box protein GID2-like	495.7680829	34.84151885	21.62227993	2.552162413	-0.688288863	-3.082726608	-3.83078597283254	-7.60180144333774
VIT_218s0001g09700	*VvGID2-2*	924	F-box protein GID2-like	745.2353334	81.06710334	140.8388632	161.5883806	0.796856989	0.198277974	-3.20050757839836	-2.20537261593714
VIT_207s0104g00930	*VvGID1B*	1699	gibberellin receptor GID1B-like	1056.012332	163.7519686	92.77871026	98.04269357	-0.819646554	0.07961633	-2.68904252620159	-3.42907275112994
VIT_214s0068g01610	*VvGAI1-1*	2007	DELLA protein GAI1-like	1.38778E-17	0.01343498	0.011435734	0.012049474	-0.232445147285861	0.0754212261133466	49.7821379450127	49.6251140238401
VIT_217s0000g10300	*VvGAI1-2*	1575	DELLA protein GAI1-like	0.023	0.017120003	0.014572393	0.015354473	-0.232445147285869	0.075421226113353	-0.425950938294278	-0.582974859466794
VIT_207s0005g01500	*VvGAI1-3*	1815	DELLA protein GAI1-like	0.023	0.356548815	0.227618365	0.01332413	-0.647482646564714	-4.09450377532896	3.95439384281841	-0.787592579075264
VIT_214s0006g00640	*VvGAI1-4*	2251	DELLA protein GAI1-like	2614.12558	139.455769	155.5523614	178.8983693	0.157592678778968	0.201739940940811	-4.22844892584374	-3.86911630612396
VIT_201s0011g05260	*VvGAI1-5*	2379	GAI1	640.585936	6.596490292	3.357346961	2.744636262	-0.974376994345422	-0.290706672684547	-6.60154968113818	-7.86663334816815
VIT_219s0085g00540	*VvRGL1*	1593	DELLA protein RGL1-like	4.233074983	0.101559338	0.014407733	0.015180976	-2.81740764800702	0.0754212261133484	-5.38131124872597	-8.12329767061965
VIT_211s0016g04630	*VvSLR1*	1697	DELLA protein SLR1-like	1511.607182	217.9683021	84.63795078	56.03342144	-1.3647417443757	-0.595017110786993	-2.79389302622774	-4.75365188139044

miRNA

miRNA	Stone hardening	Veraison	Ripe	Target gene	Stone hardening	Veraison	Ripe	Gene
VvmiR3633a	7821	3629	1689	VvGA3ox1-3	0.022658827	0.01928699	0.020322097	VIT_209s0002g05300

VvmiR396c/d	59	85	90	VvGID1B	163.7519686	92.77871026	98.04269357	VIT_207s0104g0093<br>0			
VvmiR159c	1360	1204	787	VvGAMYB1	3.408010061	1.550778274	1.614782818	VIT_213s0067g0163<br>0			
VvmiR477	134	246	821	VvGAI1-2	0.017120003	0.014572393	0.015354473	VIT_217s0000g1030<br>0			
VvmiR319b/c/f/g/e	20	2	2	VvGAMYB1	3.408010061	1.550778274	1.614782818	VIT_213s0067g0163<br>0			
VvmiR319b/c/f/g/e	27	87	194	VvGAMYB2	2.12139163	1.164207458	0.275379136	VIT_206s0009g0248<br>0			
