# Supplementary material for: Genome-wide identification and characterization of gibberellin metabolic and signal transduction (GA MST) pathway mediating seed and berry development (SBD) in grape (Vitis vinifera L.)
Source: BMC Plant Biol. 2020 Aug 21;20:384. doi: 10.1186/s12870-020-02591-1 (PMC7441673; doi:10.1186/s12870-020-02591-1)
Supplement: Supplementary file 2 — Additional file 2: Table S2. The gene expression patterns of auxin, ABA and CK MST pathway genes in three stages of grapevine SBD. [file 12870_2020_2591_MOESM2_ESM.pdf]

Additional file 2:Table S2 The gene expression patterns of auxin, ABA, and CK MST pathway genes in three stages of grapevine SBD.

Auxin

#Gene	Abbreviation	Function	Green -RKPM	Stone hardening-RPKM	Veraison-RPKM	Ripe-RPKM	Veraison/Stone hardening Log2	Ripe/veraison log2	Stone hardening/Green log2	Ripe/Green log2
VIT_211s0016g03930	*VvYUC 2*	flavin-containing monooxygenase YUCCA2-like	1.061963108	0.04249646	0.018086303	0.01905697	-1.232445147	0.075421226	-4.6432471562059	-5.80027107737842
VIT_204s0008g04870	*VvYUC 4*	flavin-containing monooxygenase YUCCA4-like	0.023	0.096128357	0.020455899	0.021553739	-2.232445147	0.075421226	2.06332821437157	-0.0936957068009474
VIT_204s0023g01480	*VvYUC 6-1*	flavin-containing monooxygenase YUCCA6-like	11.6324849	0.236181058	0.904658392	4.201185565	1.937479854	2.215351487	-5.62212224011898	-1.46929089870471
VIT_204s0008g03920	*VvYUC 6-2*	flavin-containing monooxygenase	57.08625846	1.555615623	0.073562559	0.01937764	-4.402370149	-1.924578774	-5.19758597440072	-11.5245348970156

		YUCCA6-like								
VIT_207s0005g04800	*VvYUC 8*	flavin-containing monooxygenase YUCCA8-like	0.023	0.017751155	0.015109624	0.015920537	-0.232445147	0.075421226	-0.373720979521722	-0.530744900694235
VIT_218s0001g15190	*VvYUC 10-1*	flavin-containing monooxygenase YUCCA10-like	0.023	0.023652635	0.020132911	0.021213417	-0.232445147	0.075421226	0.0403670659282599	-0.116656855244262
VIT_207s0104g01260	*VvYUC 10-2*	flavin-containing monooxygenase YUCCA10-like	19.03193096	2.109653135	5.087867171	12.8110365	1.270055193	1.332254293	-3.17334422459401	-0.571034738571872
VIT_218s0001g15100	*VvYUC 10-3*	flavin-containing monooxygenase YUCCA10-like	0.023	0.236526352	0.120797466	0.021213417	-0.969410741	-2.509541275	3.36229516081562	-0.116656855244262
VIT_207s0104g01250	*VvYUC 10-4*	flavin-containing monooxygenase YUCCA10-like	399.5752022	32.42172957	59.33891452	80.28344455	0.872017481	0.436123981	-3.62343409148949	-2.31529262999861
VIT_200s0394g00040	*VvTAR 1-1*	tryptophan aminotransferase 1-like	22.20312408	1.30670354	4.479078257	0.696833649	1.777270008	-2.684315676	-4.08675891440054	-4.99380458256471
VIT_200s0225g00230	*VvTAR 1-2*	tryptophan aminotransferase 1-like	0.023	0.016613681	62.90101915	19.63868323	11.88649593	-1.679385192	-0.469262109567611	9.73784862426792
VIT_217s0000g08990	*VvTAR 2*	tryptophan aminotransferase-related protein 2-like	107.8253633	6.477222732	0.598735264	0.89373047	-3.435385206	0.577921567	-4.0571774117676	-6.91464105108121
VIT_218s0157g00090	*VvTAR 4-1*	tryptophan aminotransferase-related protein 4-like	392.1757499	6.745195807	1.628172435	0.12038977	-2.050606824	-3.757468788	-5.86149619529246	-11.6695718076439
VIT_218s0157g00170	*VvTAR 4-2*	tryptophan aminotransferase-related protein 4-like	35.9449642	6.711783072	2.671755708	1.826849534	-1.328907987	-0.548430288	-2.42102167391412	-4.29835994870268
VIT_218s0157g00140	*VvTAR 4-3*	tryptophan aminotransferase-related protein 4-like	0.023	0.66577788	0.366690927	2.002102859	-0.86047637	2.448879622	4.85533517209574	6.44373842383762
VIT_218s0157g00130	*VvTAR 4-4*	tryptophan aminotransferase-related protein 4-like	6.347181498	0.019082805	0.016243113	0.017114858	-0.232445147	0.075421226	-8.37769900120738	-8.5347229223799
VIT_218s0001g08090	*VvAux*	Aux/IAA protein	3416.437625	397.5722273	314.6251718	104.9379417	-0.337582881	-1.584097701	-3.10320390255464	-5.02488448421951
VIT_208s0007g02030	*VvAux 1*	AUX-1-like protein	935.506965	9.182941878	1.101503424	1.450774508	-3.059482435	0.397349321	-6.67064817000177	-9.33278128420623

VIT_207s0005g02550	*VvIAA 4*	auxin-responsive protein IAA4-like	15.86073864	0.383147483	0.081532925	0.042954343	-2.232445147	-0.924578774	-5.37141631993051	-8.52844024110303
VIT_204s0008g00220	*VvIAA 6*	auxin-responsive protein IAA6-like	56.02919375	0.204660373	0.014517089	0.015296202	-3.817407648	0.075421226	-8.09680313272345	-11.8387895546171
VIT_211s0016g04490	*VvIAA 7*	auxin-responsive protein IAA7-like	936.5640296	41.02130499	69.77715814	24.64692038	0.766381453	-1.501347447	-4.51293233648606	-5.24789833070196
VIT_207s0005g04380	*VvIAA 11*	auxin-responsive protein IAA11-like	322.4094846	10.64426699	1.842136616	0.594184154	-2.530624639	-1.632398022	-4.92074567773661	-9.08376833953058
VIT_205s0049g01970	*VvIAA 13*	auxin-responsive protein IAA13-like	359.4067465	7.684990691	1.840652313	1.551550084	-2.061826376	-0.246506869	-5.54743016775209	-7.85576341219958
VIT_205s0020g04690	*VvIAA 14*	auxin-responsive protein IAA14-like	141.6514323	6.389574439	0.435099876	0.022922555	-3.876301337	-4.246506869	-4.47048153147747	-12.5932897373121
VIT_209s0002g05160	*VvIAA 17*	auxin-responsive protein IAA17-like	1364.6752	90.87202915	218.5089378	30.28089086	1.265784093	-2.851212737	-3.90857751877946	-5.49400616296865
VIT_211s0016g03540	*VvIAA 27-1*	auxin-responsive protein IAA27-like	2043.310686	301.5706236	208.8849516	156.049474	-0.529787338	-0.420705068	-2.76034077713692	-3.71083318377522
VIT_209s0002g04080	*VvIAA 27-2*	auxin-responsive protein IAA27-like	1482.009373	31.95733823	1.438556712	0.723431904	-4.473453247	-0.99169297	-5.53526351258457	-11.0004097291194
VIT_211s0016g05640	*VvIAA 29*	auxin-responsive protein IAA29-like	20.08899527	1.198400184	0.35702362	0.026870328	-1.74701832	-3.731933696	-4.06722375549757	-9.54617577155745
VIT_205s0020g01070	*VvIAA 31*	auxin-responsive protein IAA31-like	53.91506433	1.008725334	0.396285211	0.034796108	-1.347922365	-3.509541275	-5.74008312854117	-10.5975467678548
VIT_211s0052g00870	*VvIAA 33*	auxin-responsive protein IAA33-like	10.57542238	1.308597902	0.058624568	0.617708686	-4.480372661	3.397349321	-3.01462151751749	-4.09764485724623
VIT_212s0035g01800	*VvARF 1-1*	auxin response factor 1-like	1392.15888	30.41742579	10.51252618	20.45605783	-1.532788671	0.960418751	-5.51628199450604	-6.08865191384468
VIT_218s0089g00910	*VvARF 1-2*	auxin response factor 1-like	1264.254062	22.93344255	2.528753125	8.789494543	-3.180954832	1.797354005	-5.78468966842028	-7.16829049519433
VIT_217s0000g00320	*VvARF 2-1*	auxin response factor 2-like	2303.348582	35.09397514	16.3579752	24.63498424	-1.101229191	0.590714367	-6.03636557772297	-6.54688040217974
VIT_201s0244g00150	*VvARF 2-2*	auxin response factor 2-like	4829.733021	87.08666783	101.7922921	189.3105474	0.225104544	0.895126471	-5.79334775898053	-4.67311674338431
VIT_210s0003g00420	*VvARF 3*	auxin response factor 3-like	1531.69141	8.708219234	3.536498563	1.43029605	-1.300056053	-1.306007881	-7.45853222296702	-10.0645961569302
VIT_206s0004g03130	*VvARF 4*	auxin response factor 4-like	905.9091556	11.66591287	1.949432224	0.401182732	-2.581173302	-2.280722584	-6.2789952751497	-11.1408911607786
VIT_218s0001g13930	*VvARF 5-1*	auxin response factor 5-like	1126.835661	5.297633753	4.576802797	11.49417789	-0.211007977	1.328491246	-7.7327133035124	-6.61523003440718
VIT_211s0065g00310	*VvARF 5-2*	auxin response factor 5-like	1814.984728	6.648092903	1.394196374	4.295960531	-2.253506763	1.623546959	-8.09280115063364	-8.7227609547932
VIT_200s1203g00010	*VvARF 6-1*	auxin response factor 6-like	69.77103488	6.973449345	2.110484455	0.972891181	-1.724298244	-1.117223852	-3.32268388070125	-6.16420597614584
VIT_212s0028g01170	*VvARF 6-2*	auxin response factor 6-like	1056.012332	10.34235775	1.056933814	3.961239237	-3.290608173	1.906066798	-6.67391775699245	-8.05845913167929
VIT_210s0003g04100	*VvARF 6-3*	auxin response factor 6-like	2811.796664	20.61122829	2.373259107	1.804251071	-3.118488958	-0.395469508	-7.09191797642239	-10.6058764423102
VIT_204s0079g00200	*VvARF 8*	auxin response factor 8-like	1438.669724	5.882657836	0.94933288	3.157753856	-2.631482168	1.733912762	-7.93405158985828	-8.83162099547281
VIT_202s0025g01740	*VvARF 9*	auxin response factor 9-like	1516.892506	14.17592864	1.670735534	1.369201264	-3.084887959	-0.287148853	-6.7415317954982	-10.1135686076416

VIT_218s0001g04180	*VvARF 17*	auxin response factor 17-like	247.357896	5.790076123	4.496770857	3.904199673	-0.364692946	-0.203862531	-5.41687382737534	-5.98542930432517
VIT_215s0046g00290	*VvARF 18-1*	auxin response factor 18-like	1639.512001	7.609473559	0.337936468	0.217600201	-4.492972698	-0.635072157	-7.75125409884861	-12.8792989530494
VIT_213s0019g04380	*VvARF 18-2*	auxin response factor 18-like	483.0833074	11.63601365	3.61931533	6.437943746	-1.684808182	0.830883164	-5.37560320296062	-6.22952822131721
VIT_206s0004g02750	*VvARF 18-3*	auxin response factor 18-like	580.3332526	7.819087315	1.774809863	2.50758244	-2.139335743	0.498632657	-6.21373756792749	-7.85444065398399
VIT_211s0016g00640	*VvARF 19*	auxin response factor 19-like	316.0670969	5.204449327	2.109514564	3.578594214	-1.302834475	0.762481914	-5.92434152295418	-6.46469408367821
VIT_207s0104g01230	*VvARF 23*	auxin response factor 23-like	9.518360476	1.23794567	0.021952672	0.023130842	-5.817407648	0.075421226	-2.94276509219461	-8.68475151408828
VIT_207s0005g00660	*VvSAUR 2*	indole-3-acetic acid-induced protein ARG2-like	2514.761506	529.4212146	2239.514343	3605.38887	2.080697994	0.686968968	-2.24793367277823	0.519733289663084
VIT_208s0040g01670	*VvSAUR 7-1*	indole-3-acetic acid-induced protein ARG7-like	0.023	0.279419732	0.059459892	0.062651023	-2.232445147	0.075421226	3.60272813771229	1.44570421653977
VIT_216s0098g01150	*VvSAUR 7-2*	indole-3-acetic acid-induced protein ARG7-like	80.34168178	8.407027905	90.2934262	216.2703802	3.42495319	1.260143229	-3.25648089460196	1.4286155244438
VIT_208s0007g03120	*VvSAUR 7-3*	indole-3-acetic acid-induced protein ARG7-like	7.404239581	0.864577287	0.045995027	0.048463517	-4.232445147	0.075421226	-3.09828473667403	-7.25530865784655
VIT_203s0038g01080	*VvSAUR 7-4*	indole-3-acetic acid-induced protein ARG7-like	0.023	0.094610541	1.610632874	0.169707334	4.089482948	-3.246506869	2.04036706592826	2.88334314475574
VIT_218s0001g13980	*VvSAUR 7-5*	indole-3-acetic acid-induced protein ARG7-like	17.97486674	0.361125055	0.409848544	0.755727971	0.182592352	0.882776148	-5.6373387444852	-4.57197024432127
VIT_219s0014g04690	*VvGH3 6-1*	indole-3-acetic acid-amido synthetase GH3.6-like	521.1376338	4.057440622	0.765050615	1.128553769	-2.406942879	0.560848053	-7.00495065012989	-8.85104547536431
VIT_212s0134g00230	*VvGH3 6-2*	indole-3-acetic acid-amido synthetase GH3.6-like	0.023	0.014567263	0.012399524	0.013064989	-0.232445147	0.075421226	-0.658904004890212	-0.815927926062734
VIT_201s0150g00300	*VvGH3 17*	indole-3-acetic acid-amido synthetase GH3.17-like	23.26018857	0.668027129	0.180924132	0.217867523	-1.884521844	0.268066304	-5.12181228886016	-6.73826782866997
VIT_214s0030g01240	*VvTIR 1-1*	protein TRANSPORT INHIBITOR	630.0152898	17.34064404	12.93990782	19.95121842	-0.422330142	0.624649515	-5.18315745483917	-4.9808380817809

		RESPONSE 1-like								
VIT_200s0181g00040	*VvTIR 1-2*	protein TRANSPORT INHIBITOR RESPONSE 1-like	364.6920696	2.123877038	1.048014541	6.101036767	-1.019041509	2.541395691	-7.42383477426448	-5.90148059282373
VIT_207s0104g01320	*VvTIR 1-3*	transport inhibitor response 1	1525.349022	22.46403532	5.425458545	4.19648148	-2.049800128	-0.370564861	-6.0853784714289	-8.50574346121475
VIT_218s0001g07120	*VvTIR 1-4*	transport inhibitor response 1-like protein	871.0260232	11.20856642	5.040333464	8.318511578	-1.15301068	0.722806227	-6.28004214779304	-6.71024660021618

ABA

#Gene	Abbreviation	Function	Green-RKPM	Stone hardening-RPKM	Veraison-RPKM	Ripe-RPKM	Veraison/Stone hardening Log2	Ripe/veraison log2	Stone hardening/Green log2	Ripe/Green log2
VIT_219s0093g00550	*VvNCED 1*	9-cis-epoxycarotenoid dioxygenase NCED1, chloroplastic-like	5051.71659	17.54651529	4.107743528	8.194166006	-2.094766529	0.996251013	-8.16944535179854	-9.26796086768021
VIT_210s0003g03750	*VvNCED 2-1*	9-cis-epoxycarotenoid dioxygenase 2	1789.615177	4.413785613	0.599517243	53.56752515	-2.880143403	6.481413586	-8.66341712462968	-5.06214694219548
VIT_205s0051g00670	*VvNCED 6*	9-cis-epoxycarotenoid dioxygenase NCED6, chloroplastic-like	41.23028775	2.788323155	0.391219065	1.923671198	-2.833349192	2.297813647	-3.8862348516903	-4.42177039611655
VIT_213s0019g01010	*VvABA 2*	xanthoxin dehydrogenase-like	424.9447532	54.63237409	133.1750148	69.8058215	1.285495419	0.353591238	-2.9594472666624	-2.60585602857384
VIT_208s0058g00470	*VvPYL 4-1*	abscisic acid receptor PYL4-like	33.83083481	11.70723505	18.44602407	32.95183392	0.655909497	0.837048875	-1.53093838920384	-0.0379800178047853
VIT_213s0067g01940	*VvPYL 4-2*	abscisic acid receptor PYL4-like	3.176031061	1.66489153	0.308074073	0.194764792	-2.434079008	-0.661544368	-0.931796834578973	-4.02742021108735
VIT_216s0050g02620	*VvPYL 8*	abscisic acid receptor PYL8-like	257.9285423	39.64518847	85.58302059	53.60545907	1.110178815	-0.674944669	-2.70175374117764	-2.26651959593848
VIT_215s0046g01050	*VvPYL 9*	abscisic acid receptor PYL9-like	1893.20751	79.21871427	75.73187205	15.90780579	-0.064940694	-2.251165737	-4.57884745345635	-6.89495388522952
VIT_202s0012g01270	*VvPYR 1*	abscisic acid receptor PYR1-like	100.4259106	40.00084575	87.52972957	24.63958365	1.129742609	-1.828795235	-1.32802913432879	-2.02708176021713
VIT_218s0001g03620	*VvPP2C 2*	probable protein phosphatase 2C 2-like	70.82809958	0.447029458	0.415099053	0.546721064	-0.106914265	0.397349321	-7.30780811842491	-7.0173730626262
VIT_208s0007g00310	*VvPP2C 4*	probable protein phosphatase 2C 4-like	249.4720253	1.752706393	0.659571519	0.910079527	-1.409983333	0.464463517	-7.15314989593285	-8.09866971191166
VIT_214s0060g01580	*VvPP2C 5*	probable protein phosphatase 2C 5-like	1369.960523	63.99029212	51.33990282	64.68933335	-0.317772487	0.33344728	-4.42013745866335	-4.40446266584912

VIT_219s0015g01920	*VvPP2C 6-1*	probable protein phosphatase 2C 6-like	218.817151	4.945858562	1.853887902	0.345911667	-1.415666971	-2.422078433	-5.46736102807361	-9.30510643277271
VIT_211s0016g03180	*VvPP2C 6-2*	probable protein phosphatase 2C 6-like	1163.832923	4.510166159	6.253875981	10.52582267	0.471570025	0.75111048	-8.01148766295904	-6.78880715796499
VIT_209s0002g03530	*VvPP2C 6-3*	probable protein phosphatase 2C 6-like	50.74387019	3.180740434	10.4194561	0.778014854	1.71184542	-3.743338459	-3.99579900672635	-6.02729204581501
VIT_202s0025g01390	*VvPP2C 8-1*	probable protein phosphatase 2C 8-like	32.77377013	2.930870014	2.525914395	2.13575296	-0.214523239	-0.242060964	-3.48314075233704	-3.93972495536847
VIT_217s0000g03100	*VvPP2C 8-2*	probable protein phosphatase 2C 8-like	130.0237211	2.349804282	1.900125711	2.318224363	-0.306445729	0.286925331	-5.79009043978114	-5.80961083720373
VIT_218s0001g05690	*VvPP2C 10*	probable protein phosphatase 2C 10-like	81.39874646	9.299504384	66.46821827	88.80155008	2.837438947	0.417920178	-3.12978084212565	0.125578282534701
VIT_218s0001g15050	*VvPP2C 11*	probable protein phosphatase 2C 11-like	343.5507771	79.82907097	45.71839074	61.36292246	-0.804139599	0.424592572	-2.10553722000054	-2.48508424732692
VIT_218s0001g10700	*VvPP2C 12*	probable protein phosphatase 2C 12-like	547.5642493	21.33029643	19.33096715	14.44920933	-0.141990196	-0.419923269	-4.68205233543223	-5.24396580050751
VIT_201s0137g00750	*VvPP2C 14*	probable protein phosphatase 2C 14-like	67.65690549	0.082838722	0.017627894	0.018573959	-2.232445147	0.075421226	-9.67371617395838	-11.8307400951309
VIT_201s0011g03910	*VvPP2C 15*	probable protein phosphatase 2C 15-like	380.548039	9.953654418	18.53825127	42.21084229	0.897206956	1.187108458	-5.25670847670432	-3.17239306314236
VIT_204s0008g04780	*VvPP2C 22*	probable protein phosphatase 2C 22-like	248.4149607	17.01000261	3.941440669	6.667357049	-2.1095884	0.758391931	-3.86829679445543	-5.21949326331694
VIT_206s0004g05460	*VvPP2C 24*	probable protein phosphatase 2C 24-like	1810.75647	22.77575702	52.14914976	138.332173	1.195144724	1.407421098	-6.31294971247772	-3.71038389004545
VIT_213s0067g01270	*VvPP2C 25-1*	probable protein phosphatase 2C 25-like	122.6242685	6.148144265	1.794255514	1.071312094	-1.776765664	-0.744006528	-4.31794970234393	-6.83872189409844
VIT_206s0004g06840	*VvPP2C 25-1*	probable protein phosphatase 2C 25-like	3308.617034	36.91087969	21.00447447	22.47367909	-0.813349431	0.097539632	-6.48603838045855	-7.20184817979315
VIT_206s0004g07360	*VvPP2C 26*	probable protein phosphatase 2C 26-like	112.0536219	4.301290539	4.724154741	4.023635713	0.135286637	-0.231556527	-4.70327588766999	-4.79954577776342

VIT_218s0122g01010	*VvPP2C 27-1*	probable protein phosphatase 2C 27-like	693.439167	18.49189002	9.062499616	13.64124511	-1.028911753	0.589994399	-5.22880473317629	-5.66772208743391
VIT_211s0118g00700	*VvPP2C 27-2*	probable protein phosphatase 2C 27-like	72.94222896	3.453517771	2.778086681	12.5256194	-0.313975033	2.172718427	-4.40061573368413	-2.54187233899834
VIT_204s0023g00690	*VvPP2C 33*	probable protein phosphatase 2C 33-like	782.2325951	23.40872253	12.55086505	25.20701539	-0.899259402	1.006038504	-5.06247954287556	-4.95570044069963
VIT_205s0020g02360	*VvPP2C 35*	probable protein phosphatase 2C 35-like	235.7301851	5.761981689	3.964753667	4.61076259	-0.539333843	0.21777415	-5.35442762042995	-5.67598731361436
VIT_208s0040g02970	*VvPP2C 38-1*	probable protein phosphatase 2C 38-like	75.05635835	1.725938501	0.257737433	0.027156985	-2.743407067	-3.246506869	-5.44252132934131	-11.4324352646786
VIT_213s0067g03500	*VvPP2C 38-2*	probable protein phosphatase 2C 38-like	190.2764058	12.74172575	7.905098701	9.858414792	-0.688705309	0.318572208	-3.90046408379253	-4.27059718546715
VIT_219s0014g02340	*VvPP2C 39-1*	probable protein phosphatase 2C 39-like	591.9609634	127.9336405	78.20710374	43.91971861	-0.710024113	-0.832430846	-2.21010636655518	-3.75256132492122
VIT_201s0150g00510	*VvPP2C 39-2*	probable protein phosphatase 2C 39-like	73.99929366	13.95494701	9.828896285	2.467908015	-0.50567532	-1.993740798	-2.40673485329101	-4.9061509719106
VIT_205s0077g00830	*VvPP2C 42*	probable protein phosphatase 2C 42-like	618.387579	16.19389903	6.727169201	7.609398877	-1.267378937	0.177782944	-5.25498904849608	-6.34458504214831
VIT_208s0007g06510	*VvPP2C 43*	probable protein phosphatase 2C 43-like	330.8660016	4.828505594	3.070106028	2.191366543	-0.653288268	-0.486457661	-6.09852649476866	-7.23827242450934
VIT_209s0070g00490	*VvPP2C 44*	probable protein phosphatase 2C 44-like	372.091522	72.70472384	36.5997488	82.04725941	-0.990215357	1.164621398	-2.35553651100327	-2.18113046952128
VIT_207s0031g02160	*VvPP2C 47*	probable protein phosphatase 2C 47-like	162.7927252	6.989480263	1.80118932	0.862662131	-1.956235353	-1.062082298	-4.5417072392786	-7.56002488961952
VIT_207s0005g02110	*VvPP2C 49*	probable protein phosphatase 2C	3421.722949	59.10886496	44.21353024	46.73727267	-0.418886586	0.080085618	-5.85520462425402	-6.19400559264535

		49-like								
VIT_212s0059g01820	*VvPP2C 52*	probable protein phosphatase 2C 52-like	725.1511056	26.30924907	22.57152642	24.18329506	-0.221066085	0.099506845	-4.784639676595	-4.90619891711271
VIT_217s0000g07010	*VvPP2C 55-1*	probable protein phosphatase 2C 55-like	34.88789951	0.44385192	0.28335208	0.696638129	-0.647482647	1.297813647	-6.29650449198461	-5.64617349109952
VIT_208s0007g05820	*VvPP2C 55-2*	probable protein phosphatase 2C 55-like	0.023	0.03638867	0.030973709	0.032636026	-0.232445147	0.075421226	0.661855442674529	0.504831521502011
VIT_218s0001g12160	*VvPP2C 55-3*	probable protein phosphatase 2C 55-like	656.4419053	28.45235763	15.41362325	23.62710152	-0.88434217	0.616236623	-4.5280472315721	-4.79615277795629
VIT_218s0001g01220	*VvPP2C 55-4*	probable protein phosphatase 2C 55-like	0.023	0.034569236	0.029425024	0.031004224	-0.232445147	0.075421226	0.587854861230754	0.430830940058233
VIT_204s0023g01400	*VvPP2C 55-5*	probable protein phosphatase 2C 55-like	372.091522	27.37274943	14.20861729	17.35993993	-0.945974186	0.28899579	-3.76484526230312	-4.4218236589903
VIT_209s0018g01140	*VvPP2C 58-1*	probable protein phosphatase 2C 58-like	501.053406	37.37544464	18.69354444	23.81853948	-0.999550602	0.349544811	-3.74480173700025	-4.39480752791537
VIT_212s0028g03310	*VvPP2C 58-2*	probable protein phosphatase 2C 58-like	1147.976953	103.5767877	58.73436372	93.72389736	-0.818423988	0.674212119	-3.47032105242194	-3.61453292127987
VIT_204s0008g07320	*VvPP2C 59*	probable protein phosphatase 2C 59-like	1290.680677	105.0338455	159.0678617	172.6945883	0.598788093	0.118580493	-3.61920591952495	-2.90183733351554
VIT_203s0038g02650	*VvPP2C 60-1*	probable protein phosphatase 2C 60-like	1328.735003	57.56896953	39.37449209	46.22529117	-0.548030075	0.231421093	-4.5286182101014	-4.8452271917941
VIT_204s0008g06720	*VvPP2C 60-2*	probable protein phosphatase 2C 60-like	1511.607182	92.38583955	94.73605489	51.59576085	0.036241855	-0.876661057	-4.03226772922403	-4.87268693107397
VIT_218s0001g09390	*VvPP2C 63-1*	probable protein phosphatase 2C 63-like	702.9527486	25.58936863	6.060400951	2.939929988	-2.078059403	-1.043631446	-4.7798112598082	-7.90150210786844

VIT_207s0129g00520	*VvPP2C 63-2*	probable protein phosphatase 2C 63-like	562.363154	2.649749887	1.302439109	1.338867547	-1.024640262	0.039797316	-7.72950207361472	-8.71434501940456
VIT_203s0091g00470	*VvPP2C 63-3*	probable protein phosphatase 2C 63-like	731.4934933	15.59491911	30.70235911	35.48997662	0.977273444	0.20906211	-5.55169705679482	-4.36536150254847
VIT_204s0008g00740	*VvPP2C 72*	probable protein phosphatase 2C 72-like	15.86073864	0.490626307	0.487219497	0.110007407	-0.010052726	-2.146971195	-5.01469155638067	-7.17171547755319
VIT_208s0058g00660	*VvPP2C 73-1*	probable protein phosphatase 2C 73-like	3.176031061	0.546000084	0.107250086	0.037668684	-2.347922365	-1.509541275	-2.54025194426767	-6.39771558358128
VIT_214s0060g02070	*VvPP2C 73-2*	probable protein phosphatase 2C 73-like	1020.072135	56.48923293	23.92044799	25.84230739	-1.239731501	0.111490481	-4.17455145549173	-5.30279247603522
VIT_205s0077g01820	*VvPP2C 73-3*	probable protein phosphatase 2C 73-like	10.57542238	1.121084043	0.954256681	0.45072808	-0.232445147	-1.082120051	-3.23774894525902	-4.55231414341801
VIT_216s0022g02210	*VvPP2C 75*	probable protein phosphatase 2C 75-like	6.347181498	3.882816595	2.448161968	0.300947672	-0.665404555	-3.024114447	-0.709012534093216	-4.39853153609276
VIT_216s0050g02570	*VvPP2C 76*	probable protein phosphatase 2C 76-like	976.7324851	26.18310452	22.65175416	27.24568847	-0.20901339	0.26640517	-5.22125540755296	-5.16386362811186
VIT_206s0004g05050	*VvCYP707A 2*	abscisic acid 8'-hydroxylase 2-like	0.023	0.061844046	0.013160274	0.665595277	-2.232445147	5.660383727	1.42700085020344	4.85493942975208
VIT_204s0023g02350	*VvCYP707A 3-1*	abscisic acid 8'-hydroxylase 3-like	102.54004	0.206094299	0.014618802	0.015403373	-3.817407648	0.075421226	-8.95866705150954	-12.7006534734032
VIT_207s0031g00690	*VvCYP707A 3-2*	abscisic acid 8'-hydroxylase 3-like	1.39E-17	1.162085027	0.216377952	0.016285047	-2.425090225	-3.731933696	56.2167156306872	50.0596917095146
VIT_218s0076g00340	*VvCYP707A 3-3*	abscisic acid 8'-hydroxylase 3-like	88.79819923	0.019109854	0.016266136	0.017139118	-0.232445147	0.075421226	-12.1819979409843	-12.3390218621568
VIT_203s0063g00380	*VvCYP707A*	abscisic acid 8'-hydroxylase 4-like	56.02919375	1.204186063	0.277024966	0.058378503	-2.119970418	-2.246506869	-5.54004850267874	-9.9065257894802

	4-1										
VIT_218s0001g10500	*VvCYP707A 4-2*	abscisic acid 8'-hydroxylase 4-like	27.48844685	0.964914661	0.015209754	1.185926994	-5.987332649	6.284874592	-4.83228023084901	-4.53473828855605
VIT_218s0001g12870	*VvCYP707A 4-3*	abscisic acid 8'-hydroxylase 4-like	1.061963108	0.03573758	0.030419508	0.01602604	-0.232445147	-0.924578774	-4.89314789289148	-6.050171814064
VIT_206s0009g01790	*VvABI5 2-1*	ABSCISIC ACID-INSENSITIVE 5-like protein 2	172.306307	9.157586309	4.2222133	7.376665474	-1.11696793	0.804969349	-4.23386430621917	-4.54586288736803
VIT_213s0175g00120	*VvABI5 2-2*	ABSCISIC ACID-INSENSITIVE 5-like protein 2-like	554.9637017	41.22665421	16.27079477	21.57195334	-1.341292659	0.406872095	-3.75074412511188	-4.68516468920068
VIT_212s0055g00420	*VvABI5 5*	ABSCISIC ACID-INSENSITIVE 5-like protein 5-like	325.5806785	17.75778117	11.91110669	14.82557087	-0.576143865	0.315780194	-4.19649185172585	-4.45685552259106
VIT_218s0001g04500	*VvABI5 7*	ABSCISIC ACID-INSENSITIVE 5-like protein 7	0.023	0.028807697	0.024520853	0.025836854	-0.232445147	0.075421226	0.324820455396961	0.167796534224441
VIT_201s0026g01690	*VvWRI1-1*	ethylene-responsive transcription factor WRI1-like	0.023	0.107640735	0.045811414	0.53097055	-1.232445147	3.534852845	2.22651838178515	4.52892607924993
VIT_213s0047g00340	*VvWRI1-2*	ethylene-responsive transcription factor WRI1-like	28.54551148	28.25189819	4.419776866	0.330022222	-2.676302361	-3.743338459	-0.014916110011043	-6.43455693012951

CK

#Gene	Abbreviation	Function	Green-RKPM	Stone hardening-RPKM	Veraison-RPKM	Ripe-RPKM	Veraison/Stone hardening Log2	Ripe/veraison log2	Stone hardening/Green log2	Ripe/Green log2
VIT_207s0104g00270	*VvIPT 3-1*	adenylate isopentenyltransferase 3, chloroplastic-like	32.77377013	6.759105781	1.315037763	2.381524133	-2.361728164	0.85678094	-2.27763734470574	-3.78258456929856
VIT_209s0070g00710	*VvIPT 3-2*	adenylate isopentenyltransferase 3, chloroplastic-like	21.14605964	0.867009779	0.098398793	0.02591993	-3.139335743	-1.924578774	-4.60819678044283	-9.67211129722386
VIT_205s0020g02630	*VvIPT 5-1*	adenylate isopentenyltransferase 5,	0.023	0.038301142	0.016300794	0.103053814	-1.232445147	2.660383727	0.735753556343064	2.16369213589171

		chloroplastic-like								
VIT_208s0040g01010	*VvIPT 5-2*	adenylate isopentenyltransferase 5, chloroplastic-like	1.39E-17	0.034702708	0.029538634	0.031123932	-0.232445147	0.075421226	51.1511920579676	50.9941681367951
VIT_208s0040g00100	*VvIPT 7*	adenylate isopentenyltransferase 7, mitochondrial-like	395.3469437	21.0937565	16.05606951	21.44556354	-0.393697274	0.41756046	-4.22823132512951	-4.2043681392358
VIT_201s0011g03640	*VvIPT*	adenylate isopentenyltransferase-like	12.68954789	7.905109645	0.092174773	0.024280417	-6.422269706	-1.924578774	-0.682783290175918	-9.02963177022845
VIT_200s0252g00040	*VvCKX1*	cytokinin dehydrogenase 1-like	10.57542238	0.306726004	0.16614336	0.175060047	-0.884521844	-0.809100618	3.73724471414082	2.92814409638861
VIT_200s2520g00010	*VvCKX3-1*	cytokinin dehydrogenase 3-like	0.023	0.175546902	0.014942395	0.251909324	-3.554373242	0.521047984	2.93215076914657	3.45319875308669
VIT_211s0016g02110	*VvCKX3-2*	cytokinin dehydrogenase 3-like	397.461073	2.628371963	0.105282195	0.027733137	-4.641836083	-6.566414857	-7.24050030293186	-13.8069151602421
VIT_207s0005g05960	*VvCKX3-3*	cytokinin dehydrogenase 3-like	0.023	0.240596595	0.014628119	0.893965018	-4.039800069	1.893602152	3.38691046006987	5.28051261196732
VIT_218s0001g13200	*VvCKX5*	cytokinin dehydrogenase 5-like	2048.596009	145.2881069	6.165449299	3.537390906	-4.558566768	-5.360087145	-3.81764699147379	-9.17773413647148
VIT_213s0158g00320	*VvCKX6*	cytokinin dehydrogenase 6-like	8.461299426	1.315317275	0.133284079	11.51585479	-3.302834475	3.130138756	-2.6854683973799	0.444670358174279
VIT_204s0008g01880	*VvCKX7*	cytokinin dehydrogenase 7-like	583.5044465	11.90196458	3.594816143	0.79463182	-1.727209839	-3.904769354	-5.6154720145735	-9.52024136866547
VIT_215s0021g01110	*VvCTK-HD 1*	cytokinin hydroxylase-like	0.023	0.055710752	0.023710246	0.024982743	-1.232445147	0.075421226	1.27632193770577	0.119298016533248
VIT_214s0006g02970	*VvCTK-HD 2*	cytokinin hydroxylase-like	12.68954789	3.633688214	0.664842474	4.538175018	-2.450350178	2.771027791	-1.80413412772967	-1.48345651484117
VIT_216s0050g00480	*VvCTK-HD 3*	cytokinin hydroxylase-like	2.118998898	1.860673969	0.4295021	0.876821224	-2.115088197	1.029617537	-0.187557551151721	-1.2730282112992
VIT_201s0011g05830	*VvARR 2-3*	two-component response regulator ARR2-like	667.0125515	24.07460526	10.23654816	25.57405965	-1.233782834	1.320951884	-4.79212986125541	-4.70496081096895
VIT_217s0000g07580	*VvARR 5*	two-component response regulator ARR5-like	165.9639191	27.815499	4.348708759	2.545610006	-2.67722999	-0.77257568	-2.57690873841702	-6.02671440881738
VIT_213s0067g03510	*VvARR 9-1*	two-component response regulator ARR9-like	0.023	0.080609878	0.068614405	0.036148423	-0.232445147	-0.924578774	1.8093227743389	0.652298853166383
VIT_213s0067g03430	*VvARR 9-2*	two-component response regulator ARR9-like	190.2764058	12.66083915	50.42725649	26.35256073	1.993830709	-0.936260579	-3.90965174550367	-2.85208161590016
VIT_208s0007g05390	*VvARR 9-3*	two-component response regulator	164.9068545	0.905990539	1.211840174	1.625117428	0.419631549	0.42334453	-7.50793966675796	-6.66496358793047

		ARR9-like								
VIT_213s0067g03490	*VvARR 9-4*	two-component response regulator ARR9-like	32.77377013	1.699858417	1.483076842	0.800392744	-0.196821238	-0.889813356	-4.26905515012113	-5.35568974340225
VIT_218s0001g02540	*VvARR 9-5*	two-component response regulator ARR9-like	321.35242	2.490901075	2.862314079	1.117011319	0.20051426	-1.357538181	-7.01134480007788	-8.1683687212504
VIT_213s0067g03460	*VvARR 9-6*	two-component response regulator ARR9-like	0.023	0.054804886	0.046649428	0.049153039	-0.232445147	0.075421226	1.25267066964112	1.09564674846861
VIT_213s0067g03450	*VvARR 9-7*	two-component response regulator ARR9-like	0.023	0.052459152	0.04465276	0.047049212	-0.232445147	0.075421226	1.18956062578648	1.03253670461397
VIT_217s0000g10100	*VvARR 11-1*	two-component response regulator ARR11-like	33.83083481	2.114271103	0.887826642	1.441137291	-1.251810472	0.698857875	-4.00010649400498	-4.55305909150863
VIT_201s0010g02230	*VvARR 11-2*	two-component response regulator ARR11-like	48.62974076	1.264135215	0.667132744	1.088418343	-0.922105027	0.706187416	-5.26561620973785	-5.48153381996393
VIT_211s0206g00060	*VvARR 12-1*	two-component response regulator ARR12-like	155.3932727	5.065898809	2.985263741	3.320227584	-0.762959864	0.153423738	-4.93896197759158	-5.54849810346161
VIT_204s0008g05900	*VvARR 12-2*	two-component response regulator ARR12-like	727.2652349	6.928903501	6.937436326	12.21804106	0.001775561	0.816538464	-6.71371073927321	-5.89539671468
VIT_213s0067g03070	*VvARR 17*	two-component response regulator ARR17-like	3.176031061	6.169142334	30.71019371	28.62829659	2.315575775	-0.101275781	0.957844910693745	3.17214490464616
VIT_204s0043g00690	*VvARR 22-1*	two-component response regulator ARR22-like	0.023	42.70894272	5.208855251	0.05717091	-3.035499932	-6.509541275	10.8586886072055	1.3136474006879
VIT_207s0005g05040	*VvARR 22-2*	two-component response regulator ARR22-like	0.023	0.061561653	0.052400727	0.055213002	-0.232445147	0.075421226	1.42039811537919	1.26337419420667
VIT_208s0007g08340	*VvLOG 1-1*	cytokinin riboside 5'-monophosphate phosphoribohydrolase LOG1-like	0.023	0.040304939	0.034307202	0.036148423	-0.232445147	-0.157023921	0.809322774338901	0.652298853166383

VIT_206s0004g02680	*VvLOG 1-2*	cytokinin riboside 5'-monophosphate phosphoribohydrolase LOG1-like	667.0125515	31.2149461	74.32171798	42.52252572	1.251546876	0.445990317	-4.41740502998672	-3.97141471292901
VIT_208s0040g01780	*VvLOG 3-1*	cytokinin riboside 5'-monophosphate phosphoribohydrolase LOG3-like	1.061963108	0.39528383	0.624858094	0.962267238	0.660639649	1.28354867	-1.42577280323031	-0.142224133016843
VIT_213s0064g00740	*VvLOG 3-2*	cytokinin riboside 5'-monophosphate phosphoribohydrolase LOG3-like	28.54551148	10.19444452	6.212827142	8.277503678	-0.714461346	-0.300515515	-1.48548074604889	-1.78599626081094
VIT_218s0001g14030	*VvLOG 3-3*	cytokinin riboside 5'-monophosphate phosphoribohydrolase LOG3-like	87.74113455	0.908435055	0.210886846	0.037034143	-2.106914265	-4.61645554	-6.5937261728508	-11.2101817126606
VIT_203s0038g03420	*VvLOG 5-1*	cytokinin riboside 5'-monophosphate phosphoribohydrolase LOG5-like	0.023	0.0367357	0.031269099	0.032947268	-0.232445147	-0.157023921	0.675548922137149	0.518525000964632
VIT_218s0001g00210	*VvLOG 5-2*	cytokinin riboside 5'-monophosphate phosphoribohydrolase LOG5-like	2.118998898	0.160182203	1.136213785	0.047887713	2.826448542	-1.741986422	-3.7255970676583	-5.46758348955197
VIT_208s0007g02480	*VvLOG 7*	cytokinin riboside 5'-monophosphate phosphoribohydrolase LOG7-like	3.176031061	0.082838722	0.035255789	0.037147919	-1.232445147	-1.157023921	-5.2607759084521	-6.41779982962461
VIT_204s0008g01040	*VvLOG 8-1*	cytokinin riboside 5'-monophosphate phosphoribohydrolase LOG8-like	139.537303	5.892483421	7.583631097	4.988861568	0.364012992	-0.240165156	-4.56563124953577	-4.80579640600854
VIT_206s0004g00590	*VvLOG 8-2*	cytokinin riboside 5'-monophosphate phosphoribohydrolase LOG8-like	7.404239581	9.375892929	36.73374963	30.17693539	1.970078168	1.686418301	0.340604516844751	2.02702281796775
