# Supplementary material for: Genome-wide identification and characterization of gibberellin metabolic and signal transduction (GA MST) pathway mediating seed and berry development (SBD) in grape (Vitis vinifera L.)
Source: BMC Plant Biol. 2020 Aug 21;20:384. doi: 10.1186/s12870-020-02591-1 (PMC7441673; doi:10.1186/s12870-020-02591-1)
Supplement: Supplementary file 3 — Additional file 3: Table S3. 64 genes expression patterns of GA, auxin, ABA and CK MST pathway in seed, berry skin, pericarp and flesh from a global transcriptomic atlas. [file 12870_2020_2591_MOESM3_ESM.pdf]

Additional file 3:Table S3 64 genes expression patterns of GA, auxin, ABA, and CK MST pathway in seed, berry skin, pericarp and flesh from a global transcriptomic atlas.

	Gene	Sample_geo_accession	Green	Stone hardening	Veraison	Ripe	Seed-V	Seed-MR	Seed-FS	Seed-PFS	BerrySkin-PFS	BerrySkin-V	BerrySkin-MR	BerrySkin-R	BerryPericarp-FS	BerryPericarp-PFS	BerryPericarp-V	BerryPericarp-R	BerryFlesh-PFS	BerryFlesh-V	BerryFlesh-MR	BerryFlesh-R
GA MST pathway	*VvbHLH137*	VitusP00103529	189.219341177	29.75695085	361.1782431	400.6015399	1312.08	1288.31	68.25	94.11	1026.00	3380.60	3145.91	3118.07	494.89	1769.39	4434.33	3534.21	389.27	3808.84	3646.64	2325.44
	VvGA20o	VitusP00174271	525.36589	41.0303	3.76603	7.59939	324.03	1605.30	204.49	251.47	107.04	431.86	653.07	222.19	206.96	56.30	183.77	520.46	120.74	1042.22	1838.29	1581.22

x1-1		2296	6542	8631	4578																
VvGA2ox1-1	VitusP00129463	763.205431952	29.05850977	1.613925255	3.519727327	523.78	165.11	51.48	76.56	216.03	102.28	114.09	164.43	187.31	304.63	135.36	316.82	307.69	91.95	97.46	103.08
VvGA2ox1-2	VitusP00046942	293.868739696	28.62844883	18.06369508	15.26383901	130.88	83.73	323.02	169.25	635.86	559.83	219.76	251.99	825.86	850.38	676.84	337.82	1012.07	555.72	389.72	367.94
VvGA2ox1-3	VitusP00129475	106.768298622	26.7744505	1.210097634	3.825125756	323.06	136.21	256.96	197.76	1213.50	217.76	132.65	145.95	397.18	987.31	442.59	320.66	987.95	369.19	148.26	126.87
VvGA2ox2-1	VitusP00207863	82.455811147	15.94669832	2.474974189	8.434611756	605.73	304.02	976.32	1257.87	138.49	64.81	111.72	108.64	278.26	131.58	52.53	81.23	360.45	81.41	61.12	54.44
VvGA2ox8-4	VitusP00212106	156.450337315	10.54231741	1.112103651	0.020203254	49.71	57.11	332.86	121.48	109.02	49.44	74.45	89.34	281.09	260.49	30.71	58.47	99.97	16.92	22.32	100.33
VvGA3ox3	VitusP00262362	4.233074983	6.279288634	0.022457454	0.023662715	10.56	14.48	46.72	7.21	36.60	15.47	8.09	31.36	23.51	190.04	22.65	28.45	112.36	12.29	11.21	12.78
VvGA3ox4-1	VitusP00191379	1227.256799916	97.39423202	11.16655863	5.287186911	1014.32	766.12	3370.71	3535.21	104.24	406.80	1164.50	1278.12	1488.10	486.49	370.04	745.16	240.92	666.94	1382.71	1292.48
VvGA3ox4-2	VitusP00185882	527.480021539	11.02940966	11.90810753	0.026031534	80.26	52.22	2764.37	1944.25	587.93	92.28	43.85	31.93	1447.56	318.29	100.27	106.59	482.29	71.81	34.35	98.96
VvGAI1-4	VitusP00067488	2614.125579844	139.455769	155.5523614	178.8983693	2303.61	1380.39	2245.53	1822.22	1052.26	1309.66	1379.92	1118.60	1547.75	1450.27	1910.54	1090.97	1059.65	1570.76	1350.97	997.63
VvGAI1-5	VitusP00145100	640.585935994	6.596490292	3.357346961	2.744636262	1350.83	1263.71	431.64	460.81	359.08	181.19	196.42	263.99	412.84	169.73	179.55	219.59	195.53	165.16	229.23	186.87
VvGAMYB1	VitusP00051049	151.165014089	3.408010061	1.550778274	1.614782818	983.23	1074.65	127.72	282.03	37.48	99.28	102.93	80.06	84.01	108.59	161.54	189.55	79.74	189.69	202.61	165.82
VvGAMYB2	VitusP00221654	73.999293655	2.12139163	1.164207458	0.275379136	135.06	150.27	86.00	75.84	49.93	32.09	45.67	49.72	43.38	58.65	39.92	47.13	46.86	34.88	39.78	40.47

	VvGID1B	VitusP00226521	1056.012331597	163.7519686	92.77871026	98.04269357	1744.44	1555.50	167.72	341.67	551.89	1906.84	2199.06	1270.95	268.25	545.13	1461.88	1932.51	426.46	2485.69	2321.99	1792.04
	VvGID2-1	VitusP00227712	495.768082887	34.84151885	21.62227993	2.552162413	128.53	55.63	672.41	583.50	108.39	54.02	50.19	181.50	397.95	168.87	90.10	191.44	86.84	42.78	66.71	140.16
	VvGID2-2	VitusP00116243	745.235333411	81.06710334	140.8388632	161.5883806	1365.97	1535.49	621.73	623.51	994.14	1649.31	1875.16	2040.80	882.13	1803.94	2166.34	2215.08	1559.08	2173.18	2214.20	2182.49
	VvSPY1	VitusP00252664	903.795026390	14.06256675	5.257435148	16.8502026	871.49	993.93	1133.54	1059.87	711.84	695.47	669.26	452.29	1047.11	574.68	873.86	994.66	995.89	848.90	1178.67	541.59
	VvSPY2	VitusP00099600	265.327994734	8.268614575	2.92150476	2.448646076	261.74	316.48	903.10	772.98	424.52	321.50	527.05	306.58	787.61	759.33	691.64	615.92	856.57	462.45	625.29	256.64
	VvSLR1	VitusP00015803	1511.607182440	217.9683021	84.63795078	56.03342144	188.03	126.64	2145.82	1302.33	1729.30	1478.32	1789.95	827.94	2352.34	2346.39	1860.13	1190.18	1821.69	1158.96	1312.33	694.09
	CTK-HD2	VitusP00067090	2.118998898	3.633688214	0.664842474	4.538175018	25.70	28.36	66.51	359.91	60.56	46.24	24.23	145.17	30.21	65.43	37.86	186.57	30.58	45.80	33.38	101.37
CTK MST pathway	*VvLOG1-2*	VitusP00216208	667.0125515	31.2149461	74.32171798	42.52252572	177.29	252.32	117.93	213.32	206.26	480.47	755.24	723.05	858.04	272.62	406.04	839.25	318.58	539.17	925.86	930.80
	VvCKX5	VitusP00119750	2048.596009	145.2881069	6.165449299	3.537390906	65.60	26.53	2560.07	1995.95	3690.63	180.28	52.66	52.12	3056.83	3420.11	1393.66	133.29	3494.92	133.55	51.87	93.18
	VvARR22-1	VitusP00184550	0.023	42.70894272	5.208855251	0.05717091	84.29	20.47	7.66	1152.88	11.82	8.52	9.52	10.64	90.47	18.01	19.69	11.81	5.97	11.99	8.11	6.77
Auxin MST pathway	*TIR1-3*	VitusP00226201	1525.349022	22.46403532	5.425458545	4.19648148	1025.17	1219.58	2154.11	6.94	1551.46	397.60	544.0567122	232.04	2184.90	1947.69	615.45	359.86	1650.91	380.00	429.9668094	32.65
	ARF6-3	VitusP00168670	2811.796664	20.61122829	2.373259107	1.804251071	426.07	429.23	2023.58	20.80	1253.23	292.48	252.260114	272.73	1804.13	1163.21	419.71	247.16	931.54	208.04	151.8729468	106.46
	VvARF18	VitusP00077384	1639.5120	7.60947	0.33793	0.21760	184.91	214.00	1063.23	5.24	753.20	27.81	50.8826	78.11	1822.22	842.86	160.92	91.64	1597.48	46.69	70.3844	57.53

	-1		01	3559	6468	0201							5308								211	
	VvARF2-2	VitusP00153473	4829.733021	87.08666783	101.7922921	189.3105474	1010.35	945.62	1149.21	32.85	1125.37	1944.67	1770.831935	1332.73	1330.62	1400.40	1600.36	1746.15	1671.27	2427.96	1949.635378	1575.91
	VvAux	VitusP00114590	3416.437625	397.5722273	314.6251718	104.9379417	793.58	552.23	3503.70	26.13	2713.99	1998.98	1181.57396	1542.00	3270.04	3269.62	2306.34	1744.68	2850.84	1452.58	1148.764082	1472.10
	VvIAA27-1	VitusP00014711	2043.310686	301.5706236	208.8849516	156.049474	223.74	272.28	1181.84	99.30	2512.77	210.42	144.5886433	215.07	2345.95	1436.22	628.71	292.40	2173.85	503.47	393.9256788	636.41
	VvSAUR2	VitusP00238068	2514.761506	529.4212146	2239.514343	3605.38887	2520.01	2848.19	156.37	33.85	1048.42	2634.95	2585.639216	3215.93	549.84	1279.12	2133.10	3134.93	765.71	2866.38	3499.904056	3649.76
	VvACO4-2	VitusP00150768	307.6105798	29.561865	112.3633143	160.3006987	1174.84	742.18	214.20	413.99	595.84	850.25	1113.189451	815.65	390.54	401.69	649.76	824.75	307.27	815.98	817.3720094	793.59
	VvACO	VitusP00013510	12.68954789	2.568000394	284.8174147	68.0356701	57.00	160.60	51.77	98.46	74.59	50.29	60.50216468	90.98	50.92	46.26	158.51	310.28	22.36	540.26	309.1170757	406.43
	VvACS	VitusP00163114	609.931062	50.64264669	3.00109008	6.180574666	887.86	259.40	596.73	893.39	1707.67	389.78	166.5093588	94.77	2101.08	1829.39	1339.78	538.51	2891.23	731.93	510.3904558	239.73
ET MST pathway	*VvACO1-1*	VitusP00204407	158.5644666	42.66787231	26.22052667	23.08323466	1273.92	1438.34	653.19	977.26	2292.30	1893.42	1867.379637	760.71	1586.84	2181.31	1630.41	1029.71	1911.94	1195.95	723.3005496	408.64
	VvACO5-1	VitusP00174439	0.023	42.3424515	50.1685365	21.5237511	74.57	55.42	90.36	62.64	56.98	71.04	69.30869295	23.05	114.84	47.34	68.29	42.72	56.60	74.50	67.55966003	18.56
	VvERF118	VitusP00146995	401.6893315	23.41412124	6.879026539	5.114394333	1335.75	1514.06	1110.29	1253.40	1097.28	582.46	503.2963456	480.94	1029.14	739.28	798.69	779.12	1259.10	811.03	716.4501988	573.06
	VvERF22	VitusP00127826	485.1974367	37.36584842	1.977097095	0.543444833	15.14	14.83	50.38	15.54	1005.87	210.82	51.62681421	61.33	608.83	546.48	192.99	65.17	534.05	44.87	16.60513353	24.67
	VvETR2-1	VitusP00205040	1048.612879	109.4807079	29.39127299	39.74662796	1487.42	561.12	977.02	1401.78	1159.38	410.86	1382.812421	695.74	1013.80	1026.18	472.96	708.98	981.73	704.88	1102.867478	618.35

	VvEIN3-3	VitusP00044360	7813.826439	278.6106173	146.9822717	167.0982233	2395.71	2073.40	2161.29	1806.60	2608.59	2121.03	2459.593237	1157.98	2222.03	2560.50	2213.25	2349.67	2712.44	1719.563636	2006.567264	965.07
	NCED2-1	VitusP00004483	1789.615177	4.413785613	0.599517243	53.56752515	243.96	63.57	986.87	97.90	622.44	558.05	215.93	73.55	2110.25	630.55	1382.16	659.83	939.06	752.79	647.33	62.24
	PYL9	VitusP00076809	1893.20751	79.21871427	75.73187205	15.90780579	1426.65	1072.04	959.40	369.59	689.71	328.22	379.52	707.09	589.31	565.60	345.41	658.28	197.23	276.50	399.53	786.69
	WRI1-2	VitusP00044292	28.54551148	28.25189819	4.419776866	0.330022222	189.24	110.05	107.34	3063.82	179.12	96.93	75.43	116.32	96.40	174.68	131.89	344.15	42.68	72.77	45.66	93.81
	NCED1	VitusP00140314	5051.71659	17.54651529	4.107743528	8.194166006	436.73	150.06	374.15	38.56	464.04	242.66	219.10	112.03	1311.97	357.47	813.07	564.46	400.60	761.87	283.91	348.03
ABA MST pathway	*VvABA2*	VitusP00051690	424.9447532	54.63237409	133.1750148	69.8058215	438.85	401.30	868.99	680.49	730.49	814.70	546.36	745.90	583.18	867.36	950.01	703.87	660.23	1387.43	671.64	1053.90
	VvABI52-2	VitusP00187095	554.9637017	41.22665421	16.27079477	21.57195334	1031.97	795.72	1593.79	1763.31	1008.03	802.06	980.12	934.69	1457.53	986.40	822.79	887.14	1172.80	1012.37	1422.43	1213.02
	VvPP2C39-1	VitusP00130612	591.9609634	127.9336405	78.20710374	43.91971861	1489.33	1549.05	821.26	1136.14	935.27	831.98	588.36	581.21	676.77	951.99	780.80	671.14	1013.05	835.93	828.80	602.01
	VvPP2C73-2	VitusP00061301	1020.072135	56.48923293	23.92044799	25.84230739	173.88	235.80	314.47	132.07	1292.49	709.46	659.86	945.58	794.05	1121.58	918.59	752.16	884.66	711.12	664.78	797.51
	VvPP2C24	VitusP00213417	1810.75647	22.77575702	52.14914976	138.332173	986.81	1103.37	945.68	488.12	456.21	2004.74	1579.63	863.60	717.44	641.52	1962.93	1747.65	539.75	1891.010774	1430.82	876.24
