# Supplementary material for: Genome-wide identification and characterization of gibberellin metabolic and signal transduction (GA MST) pathway mediating seed and berry development (SBD) in grape (Vitis vinifera L.)
Source: BMC Plant Biol. 2020 Aug 21;20:384. doi: 10.1186/s12870-020-02591-1 (PMC7441673; doi:10.1186/s12870-020-02591-1)
Supplement: Supplementary file 4 — Additional file 4: Table S4. The primers sequences of GA MST pathway genes for qRT-PCR. [file 12870_2020_2591_MOESM4_ESM.pdf]

Additional file 4: Table S4 The primers sequences of GA MST pathway genes for qRT-PCR.

Gene name	Gene ID	Forward primer sequences (5'-3')	Reverse primer sequences (5'-3')
VvGA20ox1-1	VIT_203s0063g01150	ACTTCATCCGCCTCAATCAC	GTGGGCTTAACTCGAATCCA
VvGA3ox4-1	VIT_204s0008g04920	CGTTTTCCGGTTGATGAAGT	CGTTTTCCGGTTGATGAAGT
VvGA2ox1-1	VIT_219s0140g00120	TTGAAGCCTGCCAAGAGTTT	GAAGGTACTCAACCCGACCA
VvGID1B	VIT_207s0104g00930	GGTTCCGCTCAATACTTGGA	TGGTAAACCCGGTTGAGAAG
VvGAI 1-4	VIT_214s0006g00640	CCGGTGTTTCTGGACAGATT	CAGTGTCTCGTGCCTCTCAA
VvSLR1	VIT_211s0016g04630	CGCCATAAATCAGACGGTTT	TGACTCAGCACACGTCATCA
VvActin	VIT_208s0032g00900	GCTCGCTGTTTTGCAGTTCTAC	AACATAGGTGAGGCCGCACTT
