# Supplementary material for: Genome-wide identification and characterization of gibberellin metabolic and signal transduction (GA MST) pathway mediating seed and berry development (SBD) in grape (Vitis vinifera L.)
Source: BMC Plant Biol. 2020 Aug 21;20:384. doi: 10.1186/s12870-020-02591-1 (PMC7441673; doi:10.1186/s12870-020-02591-1)
Supplement: Supplementary file 5 — Additional file 5: Table S5. The name, stage and type of data used in this study. [file 12870_2020_2591_MOESM5_ESM.pdf]

Additional file 5: Table S5 The name, stage and type of data used in this study

Name	Details
Name of data	Transcriptomic study of biological networks involving grapevine berry development
Type of data	Expression profiling by high throughput sequencing
Stage	Three developmental stages from the berry of grapevines (*Vitis vinifera*) are respectively 40 days after flowering (DAF)， 65 DAF and 90 DAF.
